# On‐Surface Friedel–Crafts Alkylation Through a Stabilized Intermediate Complex

**DOI:** 10.1002/anie.3848864

**Published:** 2026-09-29

**Authors:** Paul Schweer, Christoph J. Vonnemann, Nishanth Arumugam, Stefan M. Huber, Daniel P. Miller, Karina Morgenstern

**Affiliations:** ^1^ Physikalische Chemie I Ruhr‐Universität Bochum Bochum Germany; ^2^ Organische Chemie II Ruhr‐Universität Bochum Bochum Germany; ^3^ Department of Chemistry Hofstra University New York USA

**Keywords:** alkylation, on‐surface synthesis, reaction mechanisms, reactive intermediates, scanning probe microscopy

## Abstract

The incorporation of ${\mathrm{sp}}^{3}$‐hybridized carbon centers into planar ${\mathrm{sp}}^{2}$ carbon nanostructures is a powerful strategy in solution chemistry for tuning their electronic and structural properties. While on‐surface synthesis has demonstrated efficient coupling strategies for ${\mathrm{sp}}^{2}$ systems, $C_{sp^{3}}$─$C_{sp^{2}}$ bond formation on surfaces remains largely unexplored. Here, we report the on‐surface analogue of Friedel–Crafts alkylation on a metal surface: an intramolecular $C_{sp^{3}}$─$C_{sp^{2}}$ coupling reaction based on a specifically designed fluorinated aromatic reactant, with the metal surface itself acting as catalyst. Our reactant‐catalyst design stabilizes the key intermediate; allowing for direct real‐space imaging at the single‐molecule level. By integrating high‐resolution scanning tunneling microscopy, x‐ray photoelectron spectroscopy, and first‐principles computations, we elucidate each elementary step of the on‐surface Friedel–Crafts alkylation pathway. These results open a new route toward the controlled integration of ${\mathrm{sp}}^{3}$ centers into hybrid ${\mathrm{sp}}^{3}$─${\mathrm{sp}}^{2}$ carbon nanostructures, expanding the synthetic toolbox of on‐surface chemistry.

## Graphical abstract

On‐surface Friedel–Crafts alkylation is demonstrated on Ag(111) at the single‐molecule level. STM, XPS, and DFT calculations uncover the reaction mechanism and explain the stabilization of the key intermediate, providing insight into noble‐metal‐surface‐mediated $C_{sp^{3}}$─$C_{sp^{2}}$ coupling.

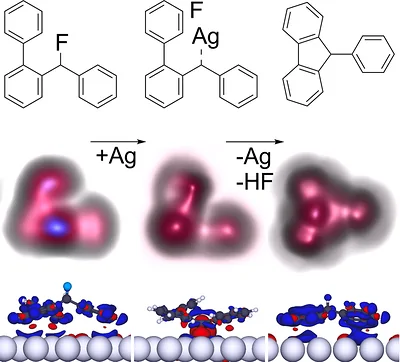

## 1 Introduction

On‐surface synthesis has emerged as a versatile approach for constructing polycyclic aromatic hydrocarbons (PAHs) with atomic precision [1, 2, 3, 4]. It typically involves the formation of C─C bonds through intermolecular couplings or intramolecular cyclizations of molecular building blocks [5, 6, 7]. These reactions mainly involve coupling via ${\mathrm{sp}}^{2}$‐hybridized carbon atoms and are frequently based on Ullmann coupling from halogenated reactants [8].

The incorporation of ${\mathrm{sp}}^{3}$‐hybridized carbon atoms into PAHs alter the electronic properties of purely $C_{sp^{2}}$‐based nanodots and nanoribbons [9, 10]. Such structural modifications constitute an important strategy for tuning the physicochemical properties of materials relevant to nanoelectronics [11] and catalysis [12]. In the context of on‐surface synthesis, metal‐surface‐catalyzed $C_{sp^{3}}$─$C_{sp^{2}}$ coupling reactions have been rarely realized so far and only via dehydrogenative coupling between the $C_{sp^{3}}$‐H bonds of methyl groups and $C_{sp^{2}}$‐H bonds [13, 14, 15, 16].

These dehydrogenative coupling reactions require elevated temperatures (≥573 K). However, high temperatures cause low selectivity and introduce structural defects even in $C_{sp^{2}}$ nanostructures [17, 18]. Therefore, alternative on‐surface synthetic strategies are needed to mitigate negative effects associated with high‐temperature conditions.

In organic chemistry, the Friedel–Crafts alkylation represents a fundamental and widely utilized reaction among the various $C_{sp^{3}}$─$C_{sp^{2}}$ bond‐forming strategies [19]. Friedel–Crafts alkylation proceeds through ${\mathrm{sp}}^{2}$‐hybridized carbocation intermediates formed by the dehalogenation of a $C_{sp^{3}}$ atom in the presence of a Lewis acid catalyst. The carbocation then acts as an electrophile and reacts with an aromatic ${\mathrm{sp}}^{2}$‐hybridized system, whose nucleophilic π‐electrons facilitate the coupling. Subsequent deprotonation by the halogen restores aromaticity, yielding the alkylated aromatic product. In the product, the introduced carbon substituent returns to the ${\mathrm{sp}}^{3}$‐hybridized state. However, the fundamental research on the mechanism of Friedel–Crafts alkylation is limited because of the high reactivity of the intermediate [20]. Friedel–Crafts alkylation on a surface has been reported recently with a hydroxyl‐functionalized reactant [21]. However, this reaction utilized an inert highly oriented pyrolytic graphite (HOPG) surface for two‐dimensional confinement and required the addition of a Lewis acid catalyst to initiate the reaction. More importantly, this study does not report the intermediate complex, leaving the reaction mechanism unexplained.

In this article, we reveal each step of an intramolecular on‐surface Friedel–Crafts mechanism on a single‐molecule level. For this aim, we designed a molecule–surface system in which the metal surface both catalyses the reaction and stabilizes the carbocation intermediate. The stabilization of the intermediate complex enables its characterization in real space. We follow each step of the reaction using scanning tunneling microscopy (STM) and x‐ray photoelectron spectroscopy (XPS), and we explain the reaction steps based on first‐principles calculations which account for dispersion forces. The specific role of the leaving group and the metal surface in the on‐surface Friedel–Crafts mechanism is elucidated.

## 2 Results and Discussion

### 2.1 Individual Reaction Steps of the On‐Surface Friedel–Crafts Alkylation

To study the Friedel–Crafts alkylation via the coupling reaction of a $C_{sp^{3}}$ atom with a $C_{sp^{2}}$ atom on the Ag(111) surface, we designed the reactant 2‐(fluorophenylmethyl)biphenyl **1** (see Scheme 1). The design of the reactant **1** ensures that the $C_{sp^{3}}$ and $C_{sp^{2}}$ atoms are in close proximity, a prerequisite for investigating the Friedel–Crafts alkylation at the single‐molecule level. Reactant **1** is chiral because of the stereocenter at its $C_{sp^{3}}$ atom (marked by a red circle) that is bonded to a biphenyl group, a phenyl group, an F atom, and a H atom.

**SCHEME 1:**
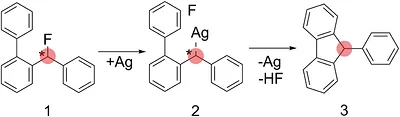
Reaction scheme of 2‐(fluorophenylmethyl)biphenyl **1** to 9‐phenyl‐9*H*‐fluorene **3** on Ag(111) through a surface‐stabilized intermediate complex **2**. The $C_{sp^{3}}$ atoms are marked by red circles.

We adsorb reactant **1** at cryogenic temperature to ensure that it adsorbs non‐dissociatively. This is true at 150 K, as evidenced by a single peak at 686.4 eV in the F 1s region of the XP spectrum (Figure 1a, purple). This binding energy is consistent with that of F atoms bonded to C atoms (686.4 eV) [22]. There are two distinct features in the C 1s region (Figure 1b): a prominent peak at 284.9 eV (blue) and a minor peak at 287.4 eV (red). The peak at 284.9 eV corresponds to C atoms within the phenyl rings (C─C/C─H, 284.7 eV to 284.9 eV) [23], while the peak at 287.4 eV is attributed to C atoms bonded to F atoms (C‐F, 287.4 eV) [24]. The peak area ratio of approximately 1:18 reflects the relative abundance of these C species within the molecule (1:18). These XPS results confirm that reactant **1** remains intact on Ag(111) at 150 K.

**FIGURE 1:**
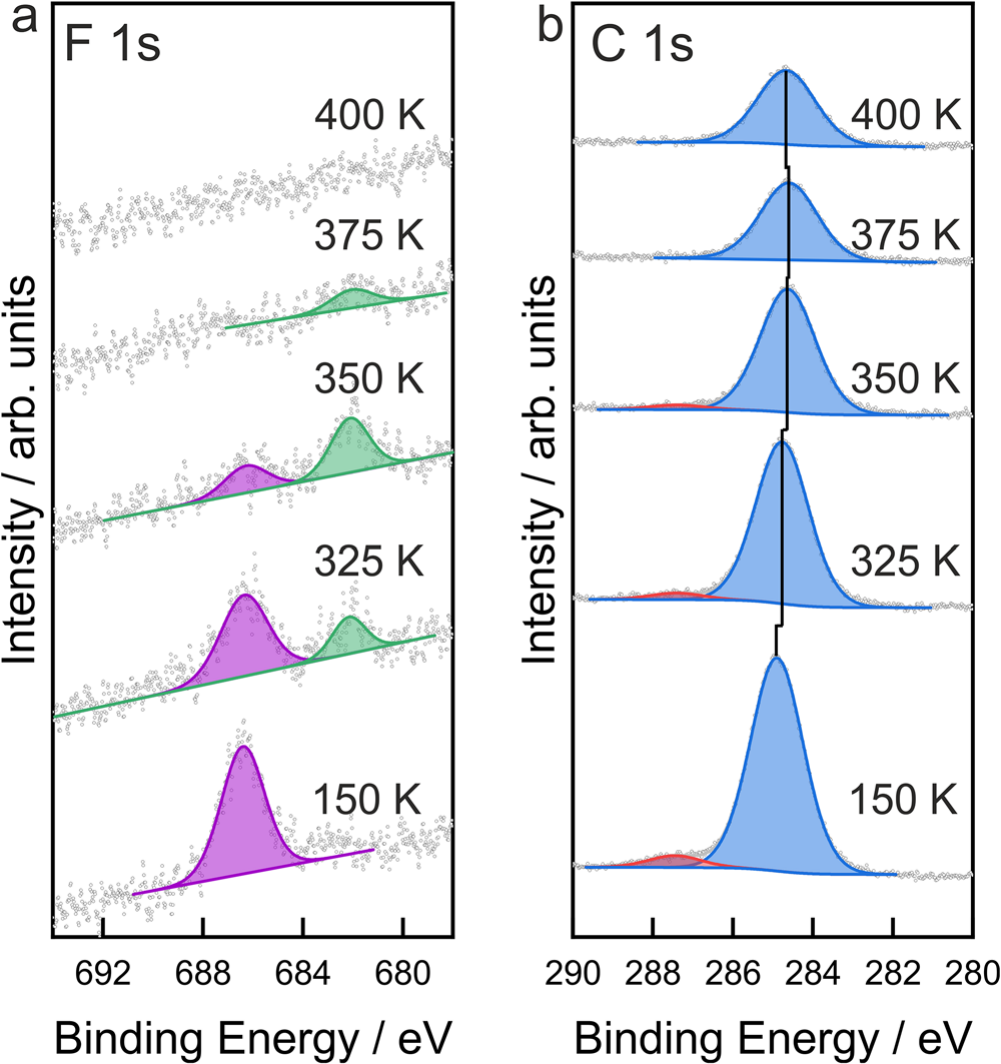
Chemical characterization of the on‐surface Friedel–Crafts alkylation on Ag(111): XP spectra of the reactant 2‐(fluorophenylmethyl)biphenyl after adsorption at 150 K and annealing at the indicated temperatures. (a) F 1s spectra with the fits to the F─C peaks and F‐Ag peaks in purple and green, respectively. (b) C 1s spectra with the fits to the C─C/C─H peaks and C─F peaks in blue and red, respectively.

We explore the adsorption structure of reactant **1** in real space upon its adsorption at 150 K. It self‐assembles into a complex superstructure (Figure 2a). This superstructure consists of extended, interconnected rows that form networks with rhomboidal pores (for the unit cell characterization, see the Supporting Information, Section S5). Since the adsorption is non‐dissociative according to the XPS data (Figure 1a, bottom), the self‐assembly is driven by non‐covalent interactions. Notably, the subsequent coupling is an intramolecular reaction localized at the center of the molecule and is therefore governed by the adsorption geometry of the individual molecule rather than by the supramolecular arrangement.

**FIGURE 2:**
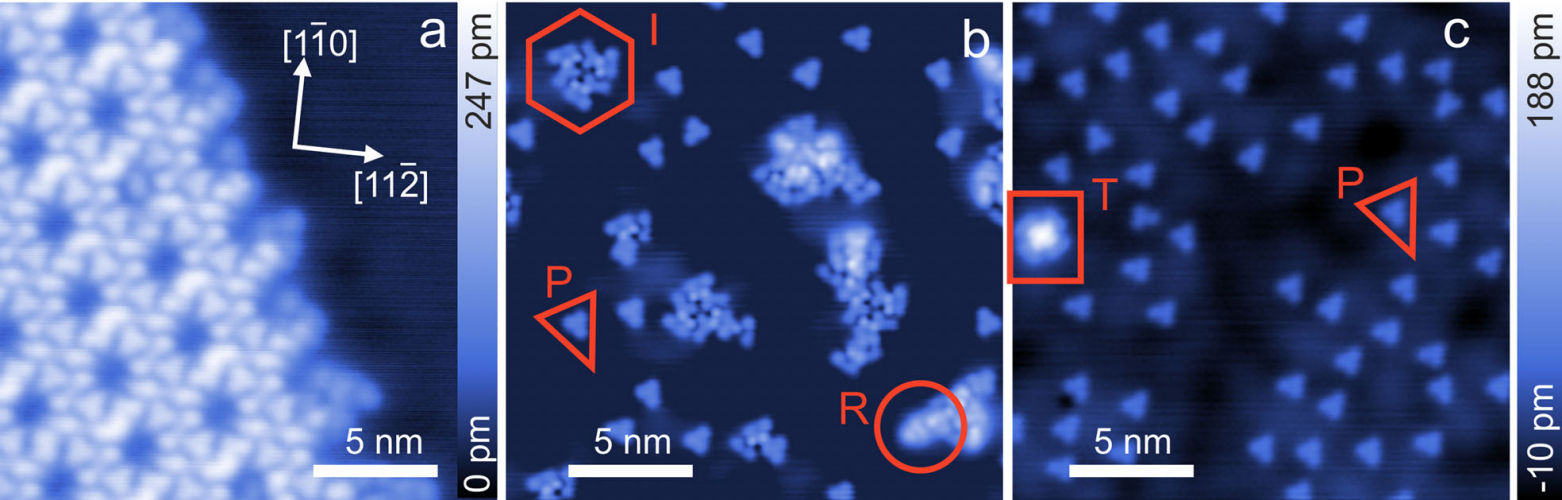
Structural characterization of the on‐surface Friedel–Crafts alkylation: (a–c) STM images after deposition at 150 K (a), after annealing at 350 K (b), and after annealing at 400 K (c). The surface orientation shown in (a) was determined from STM images with atomic resolution. In (b and c) a cluster of reactants R is marked by a circle, a cluster of intermediate complexes I by a hexagon, a product molecule P by a triangle, and a product tetramer T by a square. The images were recorded at (a) 100 mV, 5 pA, (b) 100 mV, 20 pA, (c) 100 mV, 10 pA.

To characterize an individual reactant, we isolate single molecules from the superstructure through lateral manipulation (see the Supporting Information, Section S3). Two different molecules are isolated. One molecule is imaged as a triangular protrusion in STM images (Figure 3a). In the Laplace‐filtered image, the molecule has a scalene triangular shape with a maximum located in the middle of the longest side of the triangle (Figure 3d). For the apparent height profile see the Supporting Information, Section S6. The structure of reactant **1** on Ag(111) is optimized with DFT to correlate these STM characteristics with the molecular structure (Figure 3g). The optimized structure of reactant **1** yields a geometry distinct from that in the gas phase (see the Supporting Information, Section S8). The phenyl groups rotate to maximize their π interaction with the surface, consistent with the known tendency of benzene to adsorb parallel to the Ag(111) surface [25]. The fluorine atom points toward the vacuum (Figure 3j). The calculated structure reflects the symmetry and size of the protrusion in Figure 3a fully (for a superposition of the calculated structure to the STM image see the Supporting Information, Section S4). Thus, the highest protrusion in the STM image reflects the position of the fluorine atom.

**FIGURE 3:**
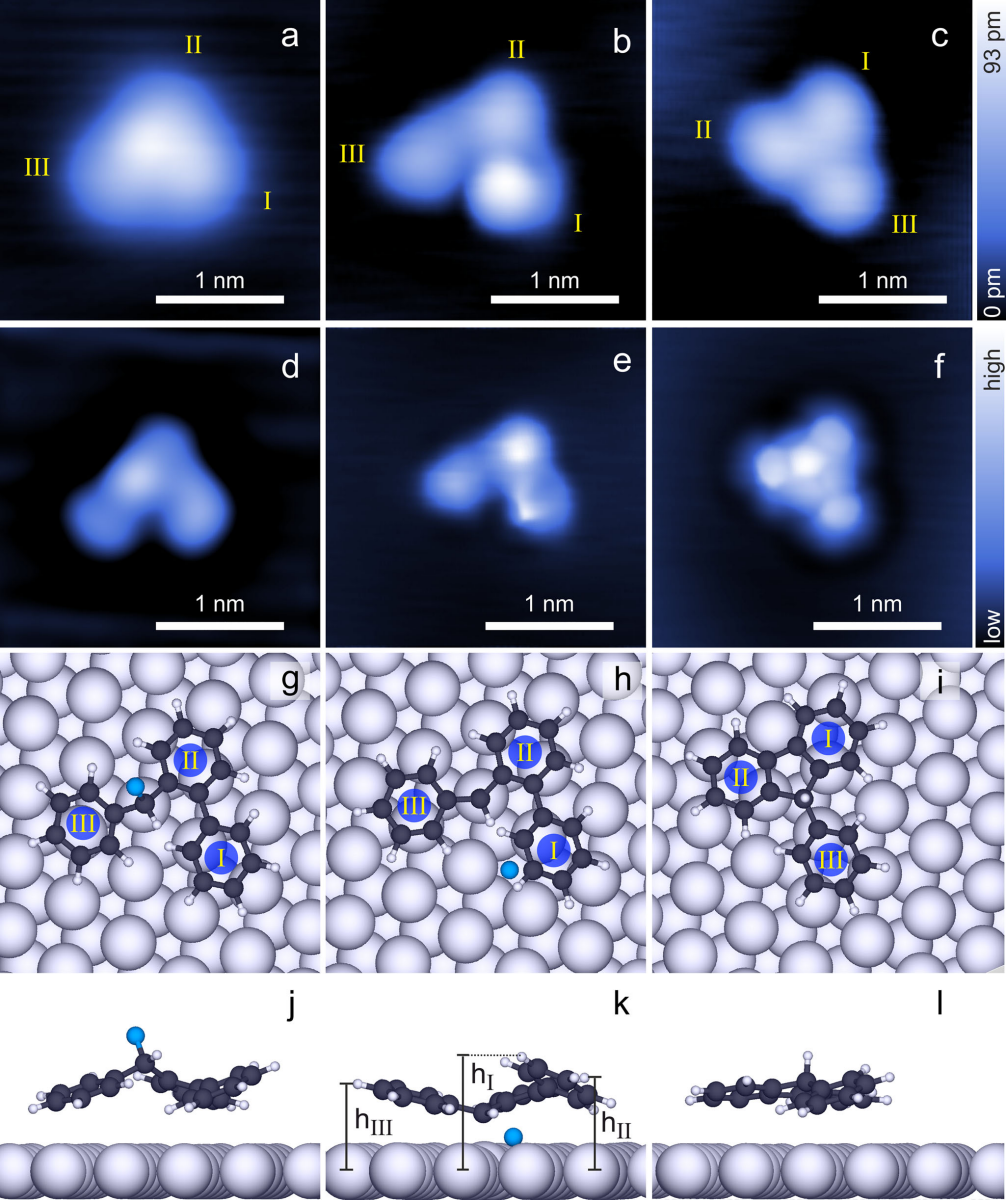
Reactant, intermediate complex and product of the on‐surface Friedel–Crafts alkylation: (a–c) Single molecule images after deposition at 150 K (a), after annealing at 350 K (b), and after annealing at 400 K (c). (d) is the Laplace filtered image of (a). (e and f) are images measured with a CO tip in constant height mode. (g–i) Top views and (j–l) side views of the calculated geometries of reactant **1** (g and j), intermediate **2** (h and k), and product **3** (i and l). Silver, black, white, and blue spheres represent Ag, C, H, and F atoms, respectively. (a) 50 mV, 10 pA, (b) 10 mV, 10 pA, and (c) 10 mV, 125 pA.

The second molecule is identified as the reactant with the F atom pointing toward the surface and phenyl ring III pointing away from the surface (see the Supporting Information, Section S3 for details). This reactant is 0.31 eV less stable than the structure with the F atom pointing away from the surface.

After having characterized the reactants, the sample is annealed to initiate the reaction. There are no significant changes in the XP spectra induced by annealing up to 300 K (not shown). Annealing at 325 K leads to an additional peak at 682.0 eV in the F 1s region of the XP spectrum (Figure 1a, green). It also causes the intensity of the F‐C peak to decrease. This change is even more pronounced upon annealing at 350 K. The peak at 682.0 eV corresponds to that of F atoms adsorbed on Ag(111) (682.0 eV) [26]. It reveals the cleavage of F‐C bonds. This interpretation is further corroborated by a decrease in intensity of the C‐F peak at 287.4 eV in the C 1s region (Figure 1b, red). Moreover, the C‐C/C‐H peak is shifted by 0.2 eV to lower binding energy (Figure 1b, blue). Such shifts toward lower binding energies are indicative of an increased core‐hole screening due to charge transfer to the molecule [27]. Thus, the C‐F bond is cleaved upon annealing at 325 K and above.

We identify the structure of intermediate complex **2** in real space on a sample with the highest intensity of the F‐Ag peak in the XP spectrum (Figure 1a), that is, upon annealing at 350 K. Indeed, the chemical change is accompanied by a significant structural change of both the superstructure and the individual molecules (Figures 2b and 3b). There are three distinctly different structures, small ordered clusters (red hexamer in Figure 2b), isolated single molecules (red triangle), and disordered clusters (red circle).

The isolated species on the surface after annealing at 350 K are attributed to product **3** (Figure 2b, P). It is confirmed by experiments at higher temperatures (see below). Since the XPS data indicate the presence of residual reactants, these are most likely situated within the disordered clusters (Figure 2b, R).

Here, we concentrate on the small ordered clusters (Figure 2b, I). Lateral manipulations reveal that the small ordered clusters contain six individual molecules (Figure 4). In contrast to reactant **1**, the intermediate complex **2** consists of three well separated protrusions of different apparent heights while forming a similar scalene triangle (Figure 3b). Protrusion I has an apparent height of 92 pm, protrusion II of 69 pm, and protrusion III of 52 pm (for an apparent height profile see the Supporting Information, Section S6). The maximum of intermediate complex **2** is located at another position than in reactant **1**. In the reactant, the maximum is located in the middle of the longest side of its scalene triangle. The maximum of intermediate complex **2** is located closer to the corner of its scalene triangle. The altered imaging characteristics indicates a structural change induced by the C‐F bond dissociation.

**FIGURE 4:**
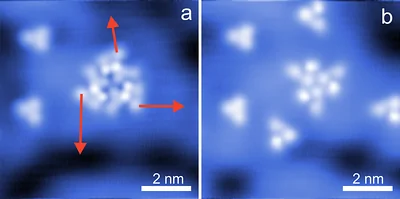
Identification of individual intermediate complexes **2** after annealing at 350 K: (a and b) STM images before (a) and after (b) lateral manipulations along the manipulation paths marked by red arrows in panel (a). The manipulation resistance was 0.83 GΩ. The images were recorded at 100 mV and 10 pA.

We turn to the calculated C‐F bond dissociated structure to interpret the imaging characteristics of intermediate complex **2** (Figure 3h). Phenyl rings II and III orient their π‐systems parallel to the surface, similar to the orientation of the phenyl rings in reactant **1** in Figure 3g. The two phenyl rings II and III bonded to the $C_{sp^{3}}$ atom are located at slightly different heights above the surface ($h_{3}$ = 367 pm and $h_{2}$ = 392 pm), whereas phenyl ring I resides at a considerably larger distance of $h_{1}$ = 480 pm (Figure 3k). The higher height of phenyl ring I results from an out‐of plane tilting caused by the presence of the fluorine at a hollow site below it (Figure 3k). This tilted phenyl ring I is bond‐resolved in Figure 3e, matching the DFT calculated structure. The $C_{sp^{3}}$ atom is interacting with a Ag(111) surface on‐top site at a Ag‐C distance of 238 pm. This distance is much shorter than the distance of 461 pm of the same C atom in reactant **1** to the Ag surface. It indicates a strong interaction between the $C_{sp^{3}}$ atom in the intermediate complex **2** and the Ag(111) surface.

The charge transfer from the surface to the intermediate complex **2** is ‐0.23 e, which is much higher than the charge transfer of ‐0.02 e to the reactant **1**. This is in good agreement with the ‐0.2 eV binding energy shift of the C‐C/C‐H peak in the XP spectrum upon annealing at 350 K (Figure 1b). However, phenyl ring I is slightly positively charged. It leads to an electrostatic attraction between phenyl ring I and the ‐0.71 e charged F atom in the fcc hollow site below phenyl ring I (for details, see the charge density difference (CDD) analysis in the section below). If the F atom is located far away from the organic moiety, the system is 0.12 eV less stable. The calculations therefore indicate that the cleaved F atom remains in the vicinity of the organic moiety after C‐F bond dissociation. It is fully consistent with the apparent height of phenyl ring I in the regular image (Figure 3b) and the features in bond‐resolved image (Figure 3e). In addition, it is in agreement with the fact that individual F atoms are not released during the manipulation experiments at 5 K. A strong interaction between the fluorine and the organic moiety is further corroborated by first‐principles molecular dynamics (FPMD) simulations at 375 K (see the Supporting Information for the movie). At this temperature the intermediate complexes **2** persists in the experiment. The diffusion of the organic moiety is coupled with the F atom.

The calculated structure reflects the symmetry and size of the protrusions identified as the intermediate complex **2** well (for a superposition see the Supporting Information, Section S4). Thus, the two circular protrusions of lower apparent height in the STM image (Figure 3b) are assigned to the two phenyl rings II and III that are bridged by the $C_{sp^{3}}$ atom. The most relevant result is that the intermediate complex has the F atom in close vicinity to the activated $C_{sp^{3}}$ atom.

Reactant **1** fully converts upon annealing at 375 K. At this temperature, there is only a low intensity F‐Ag peak at 682.0 eV but no F‐C peak at 686.4 eV in the F 1s XP spectrum (Figure 1a). The remaining peak indicates remaining intermediate complex **2**. Annealing at 400 K results in a featureless F 1s spectrum. The absence of any F related peaks indicates fluorine desorption. However, desorption of atomic fluorine at 400 K is unlikely because of its high binding energy of ‐4.319 eV, according to our calculations. As fluorine desorption from Ag(111) has not yet been studied, we relate it to the desorption of another halogen, Cl, from the same surface [28]. At a lower adsorption energy of ‐3.317 eV, atomic chlorine desorbs at 780 K from Ag(111) [29]. Because product formation requires deprotonation following C‐C coupling, it is plausible that HF is generated. Subsequent desorption of HF from the surface at the reaction temperature of 375 K is facilitated by its low binding energy of 0.22 eV on Ag(111). The C 1s region is characterized by a single peak at 284.7 eV, indicating only C‐C/C‐H bonds (Figure 1b).

We characterize product **3** in real space on a sample with fully converted molecules. The majority of the structures are individual protrusions of identical shape, well‐separated upon annealing at 400 K (red triangle in Figure 2c). Seldomly, small clusters are formed (red square, T). These small clusters are identified as tetramers of the product **3** (see the Supporting Information, Section S7). An individual product **3** has an isosceles triangular shape, composed of three distinct protrusions of similar apparent height (Figure 3c). For the apparent height profile see the Supporting Information, Section S6. These three protrusions are identified as three six‐membered rings and one five‐membered ring in the bond‐resolved image (Figure 3f). Note that the $C_{sp^{3}}$ atomic height above that of the six‐membered rings is not consistent with the formation of a radical. The five‐membered ring is formed by bonding the $C_{sp^{3}}$ atom to the $C_{sp^{2}}$ atom of the phenyl ring I. This bond formation yielding product **3** demonstrates a successful on‐surface Friedel–Crafts alkylation.

The DFT‐calculated structure of product **3** exactly reflects the symmetry and size of the protrusions in the STM image (for a superposition, see the Supporting Information, Section S4). A planar geometry with the fluorene moiety (benzene rings I and II) and phenyl ring III parallel to the surface is 0.19 eV less stable than the structure with phenyl ring III turned out of plane, which is the favored structure in the gas phase. The structure with the out‐of‐plane phenyl ring III exists in the tetramers. In the tetramers, an interaction between tilted out‐of‐plane phenyl rings of adjacent molecules further stabilizes this structure (for details, see the Supporting Information, Section S7). The predominance of the planar product is attributed to kinetic limitations, because flipping of the planar product to the structure with the out‐of‐plane phenyl ring III has a barrier of 0.41 eV (for the transition state structure, see the Supporting Information, Section S8).

Thus, annealing at 400 K leads to a complete transformation into the desired product, accompanied by a desorption of hydrogen fluoride from the surface.

### 2.2 Reaction Mechanism of the On‐Surface Friedel–Crafts Alkylation

Based on the unambiguous identification of reactant **1**, intermediate complex **2**, and product **3**, we elucidate the mechanistic pathway of the surface‐mediated Friedel–Crafts alkylation, in particular the importance of the mutual interactions between the fluorine, the organic moiety, and the Ag(111) surface (Figures 5 and 6).

**FIGURE 5:**
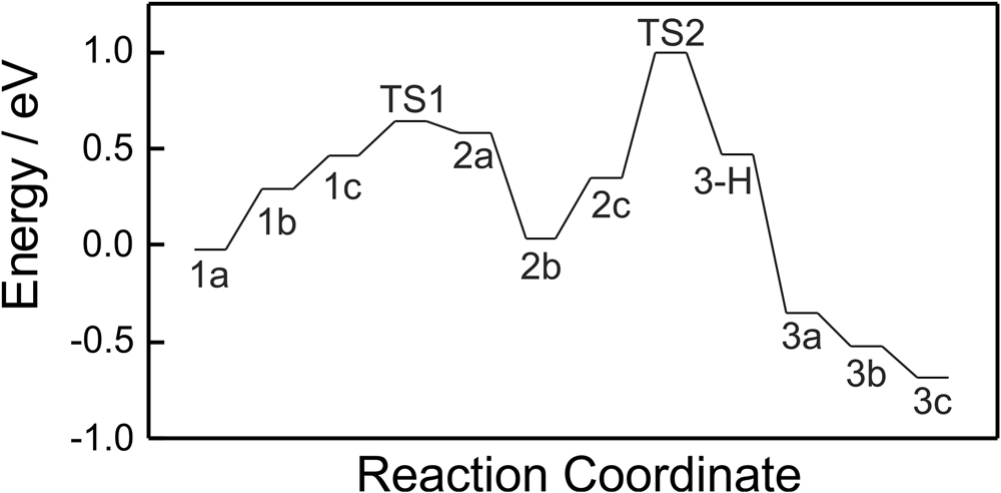
Reaction diagram of the Friedel–Crafts alkylation of reactant **1** on Ag(111). For the DFT calculated structures **1a** to **3c** see Figure 6.

**FIGURE 6:**
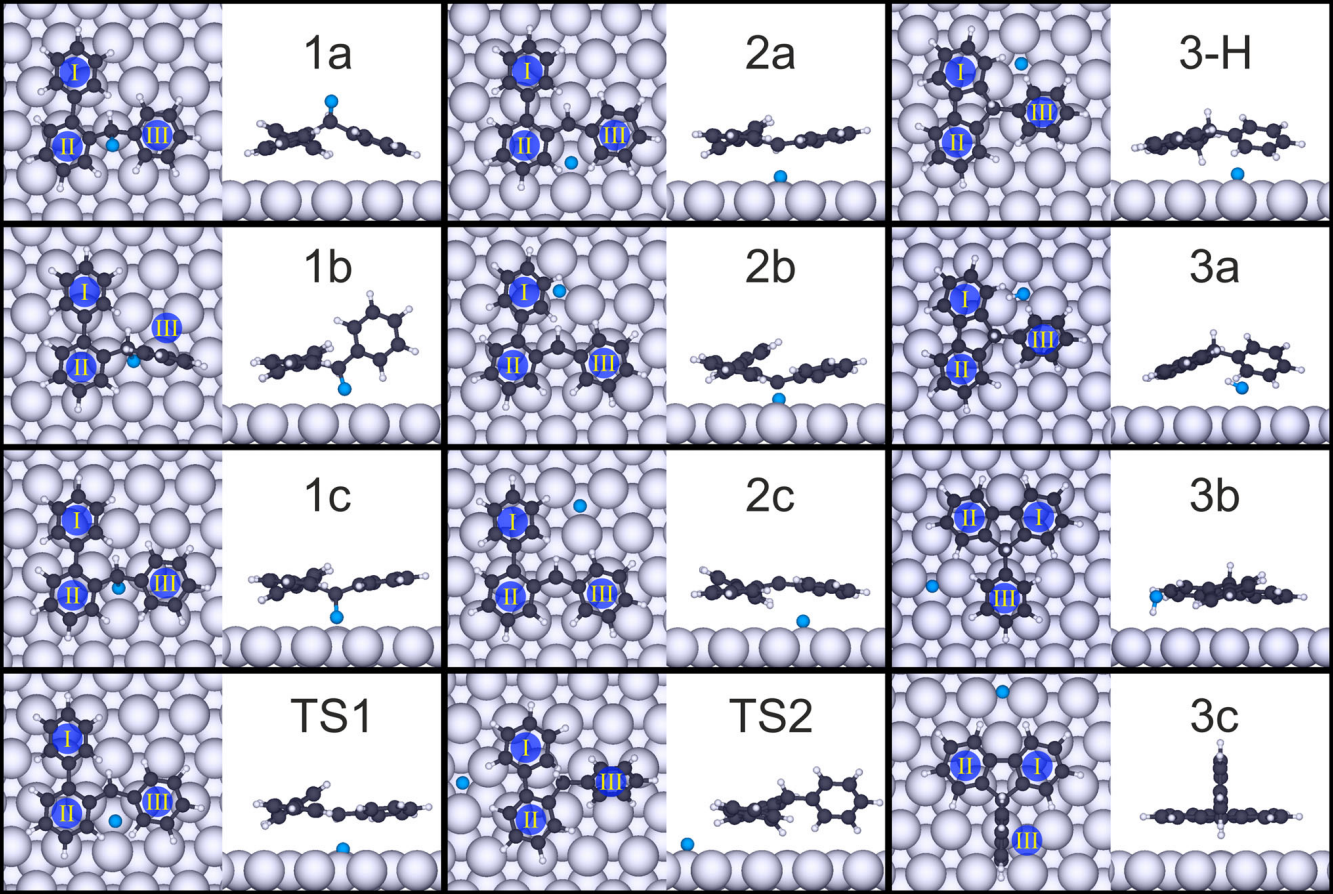
DFT calculated structures for the on‐surface Friedel–Crafts alkylation. Left columns top views and right columns side views. Silver, black, white, and blue spheres represent Ag, C, H, and F atoms, respectively. Portrayal of the reactant **1a** as chirality S instead of R in **1b** and **1c** is for simplicity reasons.

The experiments revealed two conformers upon adsorption of reactant **1** on Ag(111). For conformer **1a** the fluorine atom is oriented away from the surface and for conformer **1b** toward it (Figure 6). Conformer **1a** is energetically favored by 0.31 eV (Figure 5). However, a transition state for C‐F bond dissociation is identified only for conformer **1b**. It underlines the importance of the catalytic activity of the Ag(111) surface. Consequently, conformer **1a** must rotate about the $C_{sp^{3}}$ center to orient the fluorine atom toward the surface, simultaneously directing phenyl ring III away from the surface. The barrier for rotation about the $C_{sp^{3}}$ center is 0.56 eV. The rotation from **1b** back to **1a** is 0.25 eV. It is possible that a smaller energy pathway to rotation is necessary. This is due to the flipping of **1a** into **1b** occurring at lower temperatures than C‐F bond dissociation into forming the intermediate complex. Rotating one or two phenyl rings in **1a** prior to rotating the biphenyl to form **1b** requires 0.40 eV  or 0.29 eV, respectively, detailing a potentially lower energy pathway occurring experimentally. For the transition state structures, see the Supporting Information, Section S8.

Before reaching the transition state **TS1** for C‐F bond dissociation (Figure 6), phenyl ring III is oriented parallel to the surface. Thus, the ring rotates from its perpendicular orientation in conformer **1b** to a parallel orientation in structure **1c**. This rotation destabilizes the molecule by 0.18 eV (Figure 5). Transition state **TS1** for C‐F dissociation is located using the nudged elastic band methodology. In **TS1**, the C‐F distance is enlarged and the fluorine atom is adsorbed on the surface. Fluorine adsorbs either on a bridge or a hollow site with the bridge‐site intermediate complex being 0.09 eV less stable than the hollow‐site analogue. The hollow‐site transition state lies 0.15 eV above structure **1c** (Figure 5). Notably, the C‐F bond dissociation energy on Ag(111) is only 0.65 eV for conformer **1a**, dramatically lower than a typical gas‐phase $C_{sp^{3}}$‐F bond energy of 5.03 eV [30]. Ag(111) is, thus, an extremely efficient catalyst for $C_{sp^{3}}$‐F bond dissociation.

After completing C‐F bond dissociation, the formerly $C_{sp^{3}}$ atom hybridizes into being ${\mathrm{sp}}^{2}$ in intermediate complex **2a** (Figure 6). Furthermore, this intermediate complex **2a** is positively charged at +0.21 e. In the charge density difference (CDD) isosurface this charge depletion (blue) is especially evident on the central carbon atom (light blue arrows, Figure 7a,b). In contrast, the surface‐adsorbed fluorine atom becomes more negatively charged (‐0.71 e), compared to the fluorine in reactant **1a** (‐0.61 e).

**FIGURE 7:**
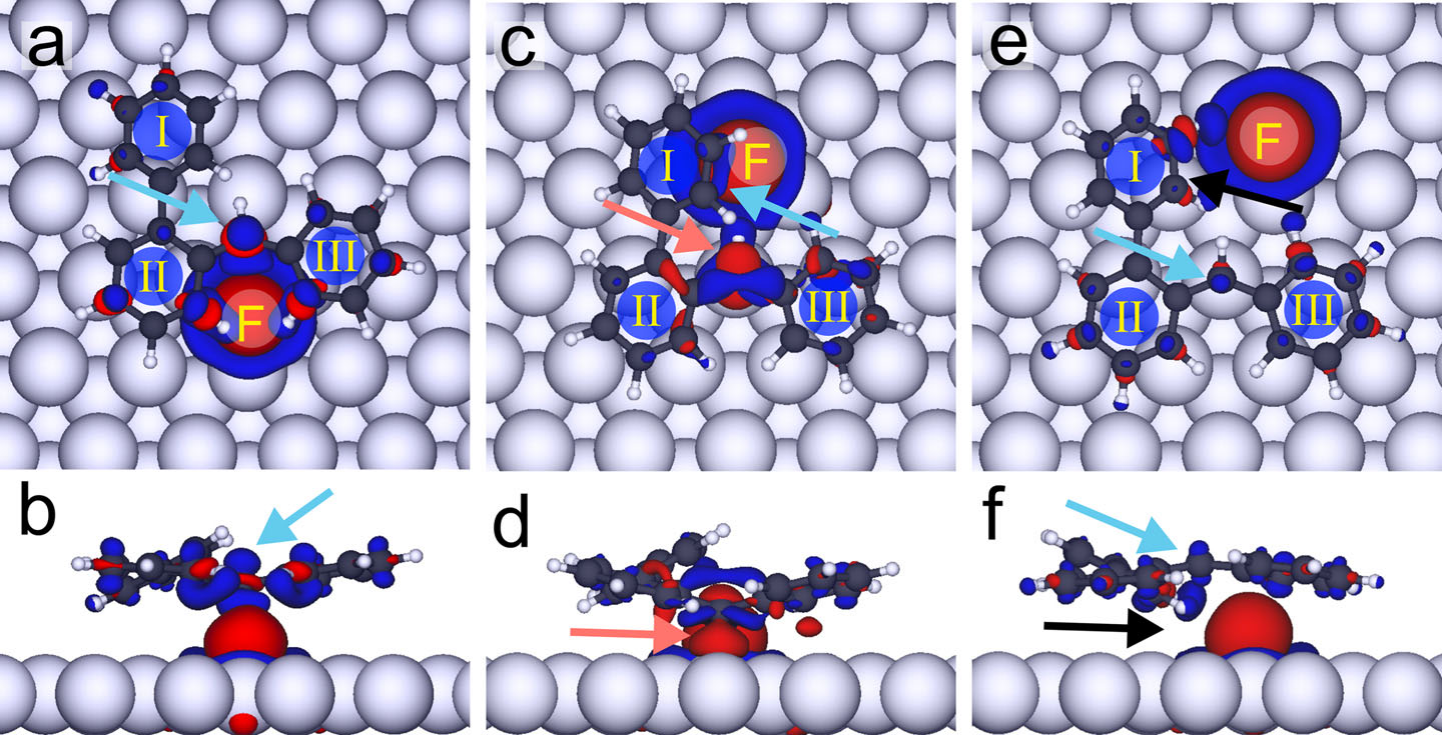
Intermediate complex stabilization through charge redistribution: (a, c, and e) Top views and (b, d, and f) side views of the CDD isosurfaces of the intermediate complexes **2a** (a and b), **2b** (c and d), and **2c** (e and f). Red and blue represent areas of charge gain and loss, respectively. The light blue and light red arrows point to the charge loss and gain discussed in the text, respectively. The black arrow points to the absence of charge gain between the central carbon atom and the surface discussed in the text. The isovalue is $\pm \;9\;e/{\mathrm{nm}}^{3}$.

In the subsequent step, the experimentally observed intermediate complex **2b** is formed. The central carbon atom of intermediate complex **2b** interacts strongly with the surface, resulting in a reduced carbon‐surface distance and rehybridization back to ${\mathrm{sp}}^{3}$. This interaction also induces charge transfer from the surface to the organic moiety, which becomes negatively charged (‐0.16 e). Both effects are reflected in the CDD isosurface of intermediate complex **2b** (Figure 7c,d). In particular, the pronounced red lobe at the $C_{sp^{3}}$ atom indicates charge gain (light red arrow, Figure 7c), while the charge gain between the central carbon atom and the surface reflects the formation of a strong carbon–surface interaction (light red arrow, Figure 7d). In addition, the organic moiety in intermediate complex **2b** interacts with the fluorine atom via phenyl ring I. This interaction leads to a charge depletion in the phenyl ring caused by the electron‐withdrawing fluorine atom evidenced by the blue lobe on the phenyl ring (light blue arrow, Figure 7c). The emergence of these carbon–surface and phenyl ring–fluorine interactions stabilize intermediate complex **2b**, lowering its energy by 0.55 eV from that of intermediate complex **2a** (Figure 5).

To assess whether this stabilization is specific to fluorine or reflects a more general property of charged leaving groups, we calculated the stability of intermediate **2b** with Cl, Br, I, and H as the leaving group. We find that **2b** is stabilized with all charged species (F, Cl, Br, and I) through C─H⋯X and phenyl⋯X interactions, while the uncharged H does not stabilize the intermediate complex, confirming that the charge of the chemisorbed leaving group is a stabilizing property (for details see the Supporting Information, Section S8).

However, the surface‐stabilized intermediate complex **2b**, in which the fluorine atom remains inserted, is not yet chemically capable of undergoing a classical Friedel–Crafts alkylation. Such a reaction has two prerequisites: a carbon electrophile and an electron‐rich aryl nucleophile in close proximity. In intermediate complex **2b**, neither condition is fulfilled. The carbon–surface interaction suppresses the positive charge that developed immediately after C‐F bond dissociation, and the chemisorbed fluorine atom withdraws electron density from phenyl ring I, reducing its nucleophilicity (Figure 7c).

Both requirements for reaction are met when phenyl ring I rotates. This rotation displaces the fluorine atom from beneath the ring and increases the molecule–surface distance, thereby weakening the carbon–surface interaction. The resulting intermediate complex **2c** is 0.31 eV less stable than intermediate complex **2b** (Figure 4). Crucially, the central carbon atom is restored to an electrophilic state, and phenyl ring I becomes more nucleophilic because the fluorine atom no longer withdraws electron density from it (Figure 7e,f). The charge loss at the central carbon is consistent with a charge back donation to the surface (light blue arrows, Figure 7e,f), as further evidenced by the absence of charge gain between the carbon and the surface (black arrow, Figure 7f). Concurrently, the disappearance of charge loss on phenyl ring I reflects the restoration of its nucleophilic character.

The C‐C coupled structure **3‐H** is 0.52 eV more stable than **TS2** but 0.44 eV less stable than intermediate complex **2b** (Figures 5 and 6). This lower stability compared to intermediate complex **2b** is due to its positive charge and the lost aromaticity of phenyl ring I. The following deprotonation restores the neutral charge and aromaticity of phenyl ring I. The nudged elastic band computations for deprotonation could not find any structure higher in energy than the C‐C coupled structure **3‐H**. This hints at a tunneling process for deprotonation. Two mechanistic pathways for deprotonation are plausible (see the Supporting Information, Section S8). In the first, the proton is transferred directly to the chemisorbed fluorine atom, which acts as a base, yielding adsorbed HF and stabilizing the structure by 0.83 eV, resulting in structure **3a** (Figures 5 and 6). In the second pathway, dehydrogenation occurs directly to the Ag(111) surface, followed by diffusion of the adsorbed hydrogen to the fluorine to form HF. Removal of the HF molecule from underneath the product further stabilizes the system by 0.17 eV, that is structure **3b** (Figures 5 and 6). The ability of chemisorbed fluorine to act as a base and desorb as HF is unique to fluorine since Cl, Br, and I are unable to activate deprotonation of the C─H entity (see Section S8 in the Supporting Information for a discussion on the role of the halogen in the reaction).

The planar product **3b** is 0.56 eV more stable than intermediate complex **2b** and 0.52 eV more stable than the reactant in its most stable structure **1a** (Figures 5 and 6). Among the product geometries, product **3c**, in which phenyl ring III is oriented out of plane, is thermodynamically the most stable. However, product **3b** is largely observed due to the energy requirement of 0.41 eV to flip the molecule as mentioned above. For a consistent comparison of total energies, the number of atoms was kept constant in all calculations. In addition, HF formation is essential to the thermodynamics of the reaction. The DFT computations reveal that fluorine is capable of acting as a base on Ag(111). Comparing the energies of chemisorbed H and F atoms to Ag(111) versus physisorbed HF revealed that HF bonding is crucial to the reaction due to its strong binding energy. Therefore, the HF molecule is included in **3b** and **3c** (Figure 6).

One of our considerations is that the C‐F dissociated structure **2a** already fulfills the prerequisites for C‐C coupling as stated above, e.g., having an electrophilic carbon atom and a nucleophilic phenyl ring. If using this structure for C‐C coupling, reaching the C‐C coupling transition state **TS2** only requires 0.41 eV instead of 0.96 eV from intermediate complex **2b**. This is a substantial energetic difference with the activation energy for C‐C coupling at 0.41 eV being lower than the 0.65 eV required for C‐F bond dissociation. Thus, the Friedel–Crafts alkylation on Ag(111) may proceed partially without forming the intermediate complex **2b**. Our experimental results suggest that this happens because 350 K yields approximately 57% intermediate complex and 43% product.

The detailed analysis of each step of the reaction mechanism demonstrates that the Friedel–Crafts alkylation, analogous to that in solution, is feasible in on‐surface synthesis. Strikingly, an unprecedented stabilization of the intermediate complex is revealed, driven by quenching of the positive charge at the carbon atom by the surface, together with a reduction in phenyl‐ring nucleophilicity by the adsorbed fluorine atom.

## 3 Conclusion

Our study establishes the Friedel–Crafts alkylation as a highly selective on‐surface reaction for the coupling of $C_{sp^{3}}$─$C_{sp^{2}}$ on a single‐molecule level, based on x‐ray photoelectron spectroscopy, high‐resolution scanning tunneling microscopy, and density functional theory which account for dispersion forces. The detailed analysis of each step of the reaction mechanism demonstrates that the intramolecular Friedel–Crafts alkylation analogous to that in solution is feasible in on‐surface synthesis. The findings establish metallic surfaces not only as effective catalysts for $C_{sp^{3}}$‐F bond dissociation but also as active participants in the stabilization of highly reactive intermediates and promotion of the Friedel–Crafts alkylation. The unprecedented stabilization of the intermediate is driven by quenching of the positively charged carbon atom together with a reduction in phenyl‐ring nucleophilicity. In more detail, the Ag(111) surface donates electron density to the $C_{sp^{3}}$ atom, shielding its electrophilicity. And, the chemisorbed F atom interacts with the phenyl ring, reducing its nucleophilicity. We suggest that stabilization of intermediates by charged leaving groups may be utilized for tailored and directed reactions on surfaces, in particular for the design of hybrid carbon nanostructures.

## Conflicts of Interest

The authors declare no conflicts of interest.

## Supporting information


**Supporting File 1**: anie74850‐sup‐0001‐SuppMat.pdf.

**Supporting File 2**: anie74850‐sup‐0002‐SuppMat.zip.

**Supporting File 3**: anie74850‐sup‐0003‐SuppMat.zip.

## Data Availability

The data that support the findings of this study are available from the corresponding author upon reasonable request.
