# Supplementary material for: On‐Surface Friedel–Crafts Alkylation Through a Stabilized Intermediate Complex
Source: Angew Chem Int Ed Engl. 2026 Sep 29;65(46):e3848864. doi: 10.1002/anie.3848864 (PMC13749274; doi:10.1002/anie.3848864)
Supplement: Supplementary file 1 [file ANIE-65-e3848864-s002.pdf]

On-surface Friedel-Crafts Alkylation through a Stabilized Intermediate Complex: Supporting Information

Paul Schweer,† Christoph J. Vonnemann,‡ Nishanth Arumugam,¶ Stefan M. Huber,‡ Daniel P. Miller,*,¶ and Karina Morgenstern*,†

†*Physical Chemistry I, Department of Chemistry and Biochemistry, Ruhr-Universität Bochum, Universitätsstraße 150, D-44801 Bochum, Germany*

‡*Organic Chemistry II, Department of Chemistry and Biochemistry, Ruhr-Universität Bochum, Universitätsstraße 150, D-44801 Bochum, Germany*

¶*Department of Chemistry, Hofstra University, 106 Berliner Hall, Hempstead, New York 11549, United States*

E-mail: Daniel.P.Miller@hofstra.edu; Karina.Morgenstern@rub.de

Contents

S1 Synthesis of the reactant 2-(fluorophenylmethyl)biphenyl **S3**

S1.1 General information about the synthesis S3

S1.2 Synthesis of 1,1'-biphenyl-2-yl-phenylmethanol S3

S1.3 Synthesis of 2-(fluorophenylmethyl)biphenyl S4

S2 Materials and Methods **S7**

S3 Separation of single reactant molecules from the superstructure **S11**

S4 Superposition of molecule models on real-space structures **S13**

S5 Superstructure characterization **S14**

S6 Apparent height profiles of the reactant, intermediate, and product **S15**

S7 Tetramers of the product **S16**

S8 Computational results **S17**

S8.1 Gas-phase structure of the reactant S17

S8.2 FPMD movies S18

S8.3 Charge Density Difference (CDD) isosurfaces S19

S8.4 Energetics of reactant, intermediate complex, product, and HF S26

S8.5 C-H deprotonation into the final product S30

S8.6 Charge distributions in the reactant, intermediate complex, and product . . S31

S8.7 Hexamers of the intermediate complex S33

S8.8 Energetics for the reaction on the surface vs gas phase S35

S8.9 Energetics for hydrogenated, chlorinated, brominated, and iodinated reactants S38

S8.10 Flipping barriers of reactant and product S42

References **S43**

S1 Synthesis of the reactant 2-(fluorophenylmethyl)biphenyl

S1.1 General information about the synthesis

Commercially available chemicals were obtained from *ABCR*, *Carbolution*, *Merck*, or *TCI* and used as received without further purification unless stated otherwise. Cyclohexane of technical grade was distilled before usage. Anhydrous diethyl ether and methylene chloride were stored over 0.3 nm sieves and further dried on an aluminium oxide column using an *MBraun MB SPS-800* solvent purification system. All other solvents and additives were dried according to literature procedures and stored over 0.3 nm molecular sieves. Air and moisture sensitive reactions were performed using standard Schlenk techniques. Column chromatography was performed using silica gel 60 M (0.04-0.063 mm, *Machery-Nagel*). Eluted products were tracked by thin-layer chromatography on fluorescent coated TLC-plates (F254, *Merck*) using a UV light at 254 nm and/or common TLC stains. Nuclear magnetic resonance spectra were recorded on an *Avance Neo-400* (^{1}H: 400 MHz, ^{13}C: 101 MHz, ^{19}F: 376 MHz) spectrometer at 25°C. All shift values are given in ppm and all coupling constants (J) in Hertz (Hz), and the multiplicity is indicated with standard abbreviations: s (singlet), br.s (broad singlet), d (doublet), t (triplet), q (quartet), quin (quintet), m (multiplet of higher or indeterminate order), dd (doublet of doublet).

S1.2 Synthesis of 1,1'-biphenyl-2-yl-phenylmethanol

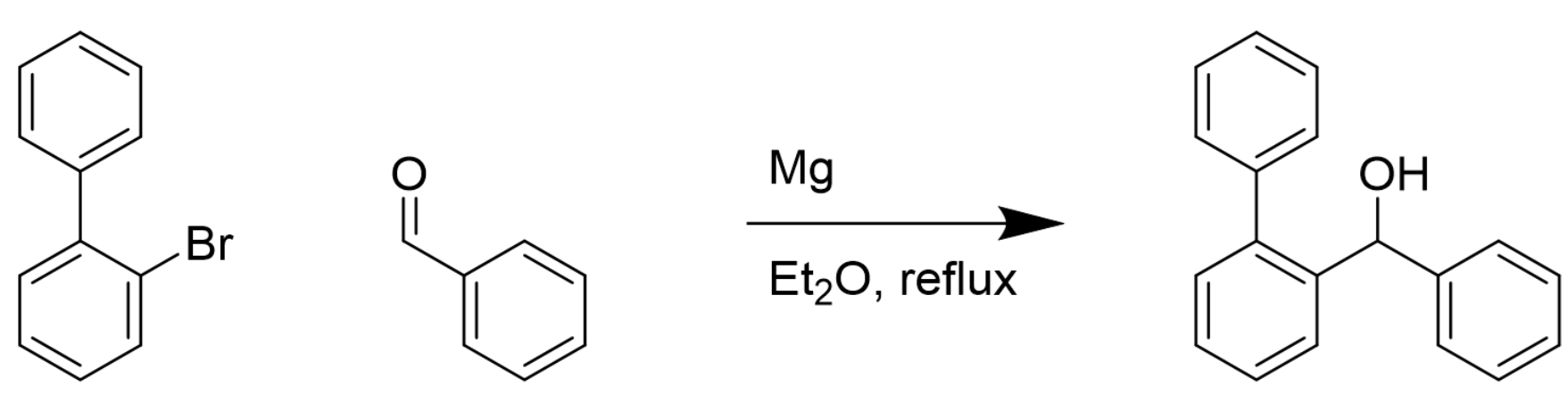

Scheme S1: Synthesis of 1,1'-biphenyl-2-yl-phenylmethanol.

1,1'-Biphenyl-2-yl-phenylmethanol was synthesized according to Scheme S1. Magnesium turnings (510 mg, 21 mmol, 1.05 eq) were activated with iodine vapor under inert atmosphere,

dried in high vacuum and suspended in anhydrous diethyl ether (10 ml). 2-Bromo-1,1'-biphenyl (3.45 ml / 4.66 g, 20 mmol, 1 eq) dissolved in anhydrous diethyl ether (40 ml) was added over 5 min and heated to reflux for 16 h. The dark solution was cooled to 0 °C and freshly distilled benzaldehyde (2.2 ml / 2.30 g, 21.6 mmol, 1.08 eq) was added dropwise under rapid stirring. The mixture was stirred for 1 h at room temperature, quenched by the addition of water and extracted with diethyl ether (3 x 50 ml). Combined organic phases were dried with magnesium sulphate, filtered, and evaporated in vacuo. The residue was purified by column chromatography (Silica, cyclohexane:ethyl acetate 90:10 with 1% triethylamine) to yield the product as a yellow solid (3.98 g, 15.3 mmol, 76%). ^{1}H NMR and ^{13}C NMR data in $CDCl_3$ was consistent with literature data.[1]

S1.3 Synthesis of 2-(fluorophenylmethyl)biphenyl

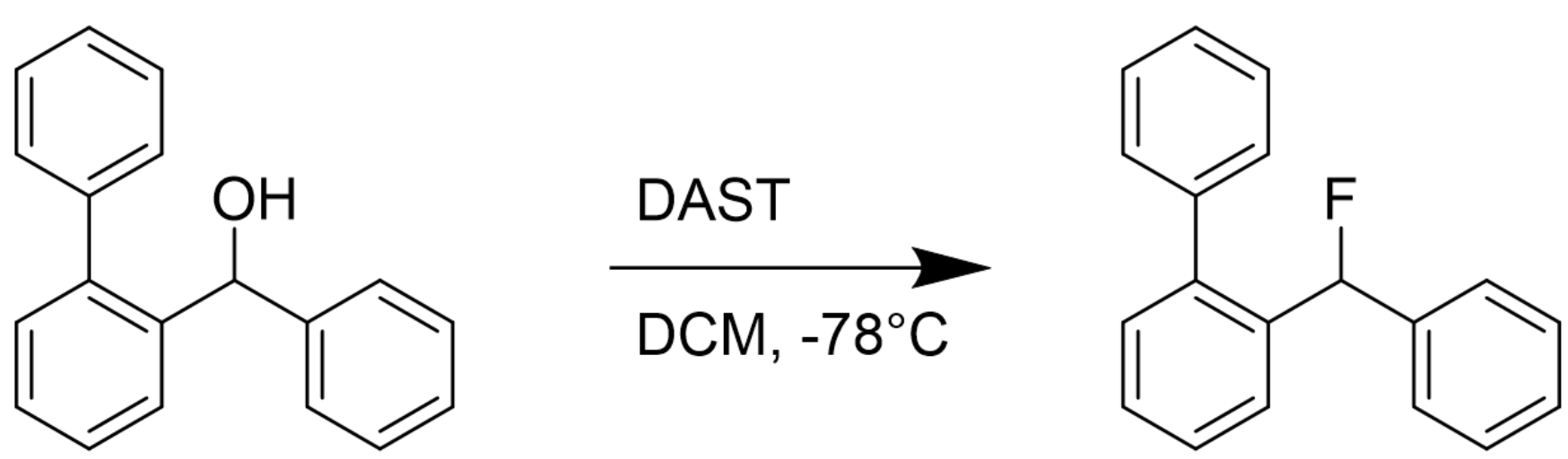

Scheme S2: Synthesis of 2-(fluorophenylmethyl)biphenyl.

2-(Fluorophenylmethyl)biphenyl was synthesized according to Scheme S2. 1,1'-Biphenyl-2-yl-phenylmethanol (1.3 g, 5 mmol, 1 eq) and di-iso-propylethylamine (DIPEA, 3.55 ml, 20 mmol, 4 eq) were dissolved in anhydrous methylene chloride (50 ml) and cooled to -78 °C. (Diethylamino)sulfur trifluoride (DAST, 1.25 ml, 10.2 mmol, 2.04 eq) was added dropwise to the solution. The resulting mixture was stirred at -78 °C for two hours. The reaction was quenched with saturated sodium carbonate solution and extracted with methylene chloride (3 x 50 ml). The combined organic phases were dried with magnesium sulphate, filtered and evaporated in vacuo. The residue was purified by column chromatography (silica, cyclohexane with 1% triethylamine) to yield the product as a colorless oil (350 mg, 27 %). ^{1}H

NMR (Fig. S1, 400 MHz, DMSO-*d6*) δ = 7.51 - 7.37 (m, 6H), 7.36 - 7.28 (m, 4H), 7.28 - 7.21 (m, 2H), 7.16 - 7.07 (m, 2H), 6.55 (d, J = 46.8 Hz, 1H) ppm. ^{13}C NMR (Fig. S3, 101 MHz, DMSO-*d6*) δ = 141.22 - 139.51 (m), 139.51 - 136.15 (m), 130.05 (s), 129.01 (s), 128.49 (dd, J = 41.7, 2.3 Hz), 128.38 (d, J = 2.5 Hz), 127.87 (s), 127.53 (s), 127.07 (d, J = 6.7 Hz), 126.32 (d, J = 6.0 Hz), 91.00 (d, J = 169.3 Hz) ppm. ^{19}F NMR (Fig. S2, 376 MHz, DMSO-*d6*) δ= -161.03 (d, J = 46.7 Hz) ppm.

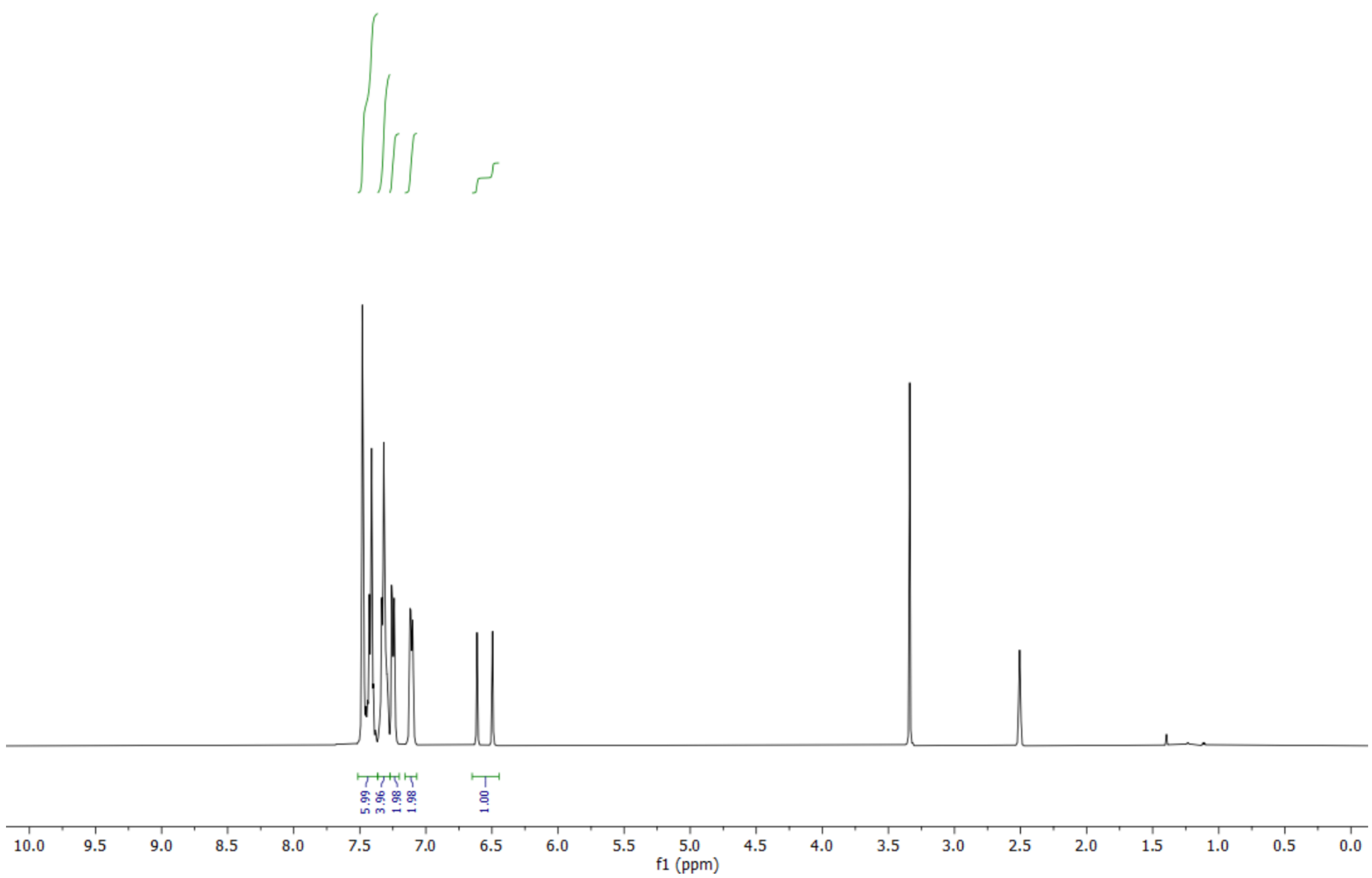

Figure S1: ^{1}H NMR Spectrum of 2-(fluorophenylmethyl)biphenyl in DMSO-*d6* measured at 400 MHz.

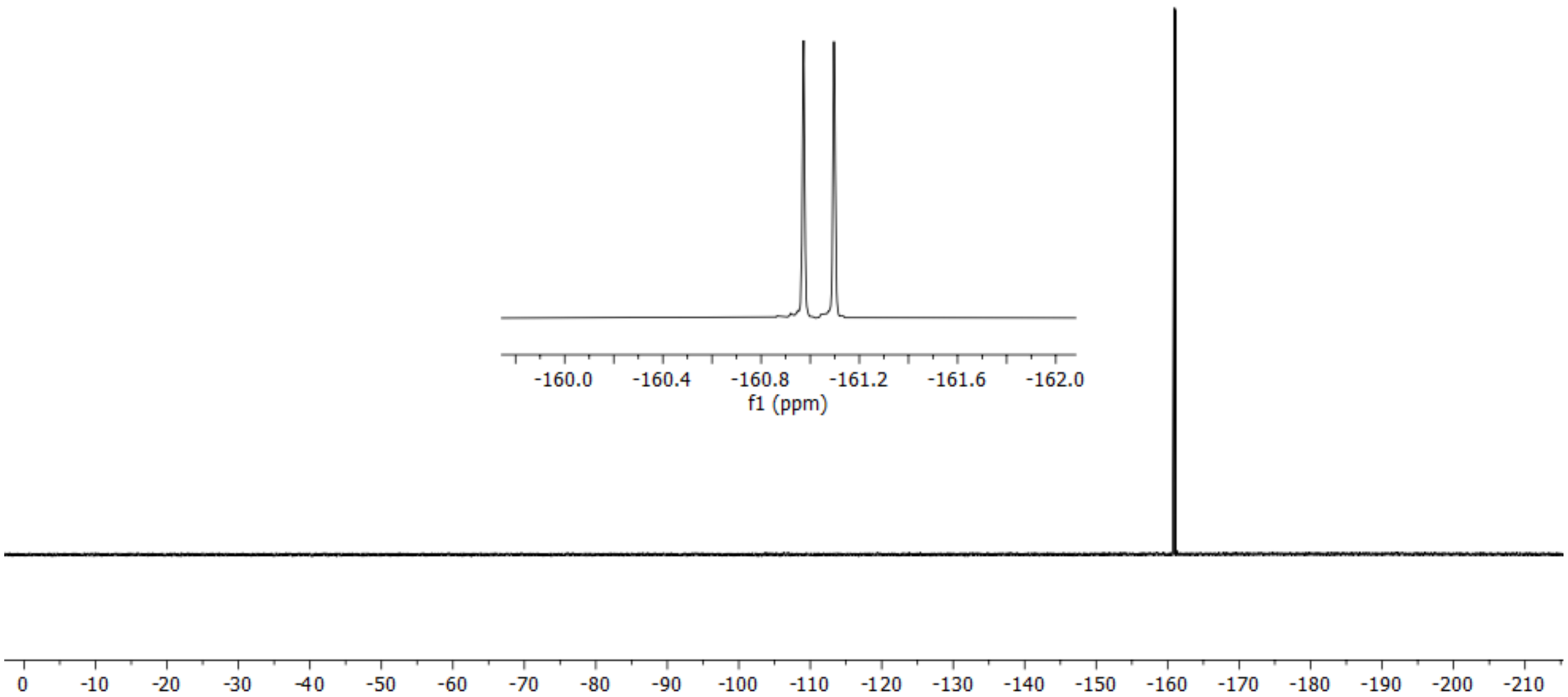

Figure S2: ^{19}F NMR Spectrum of 2-(fluorophenylmethyl)biphenyl in DMSO-*d6* measured at 376 MHz. The inset shows details of the shown signal splitting.

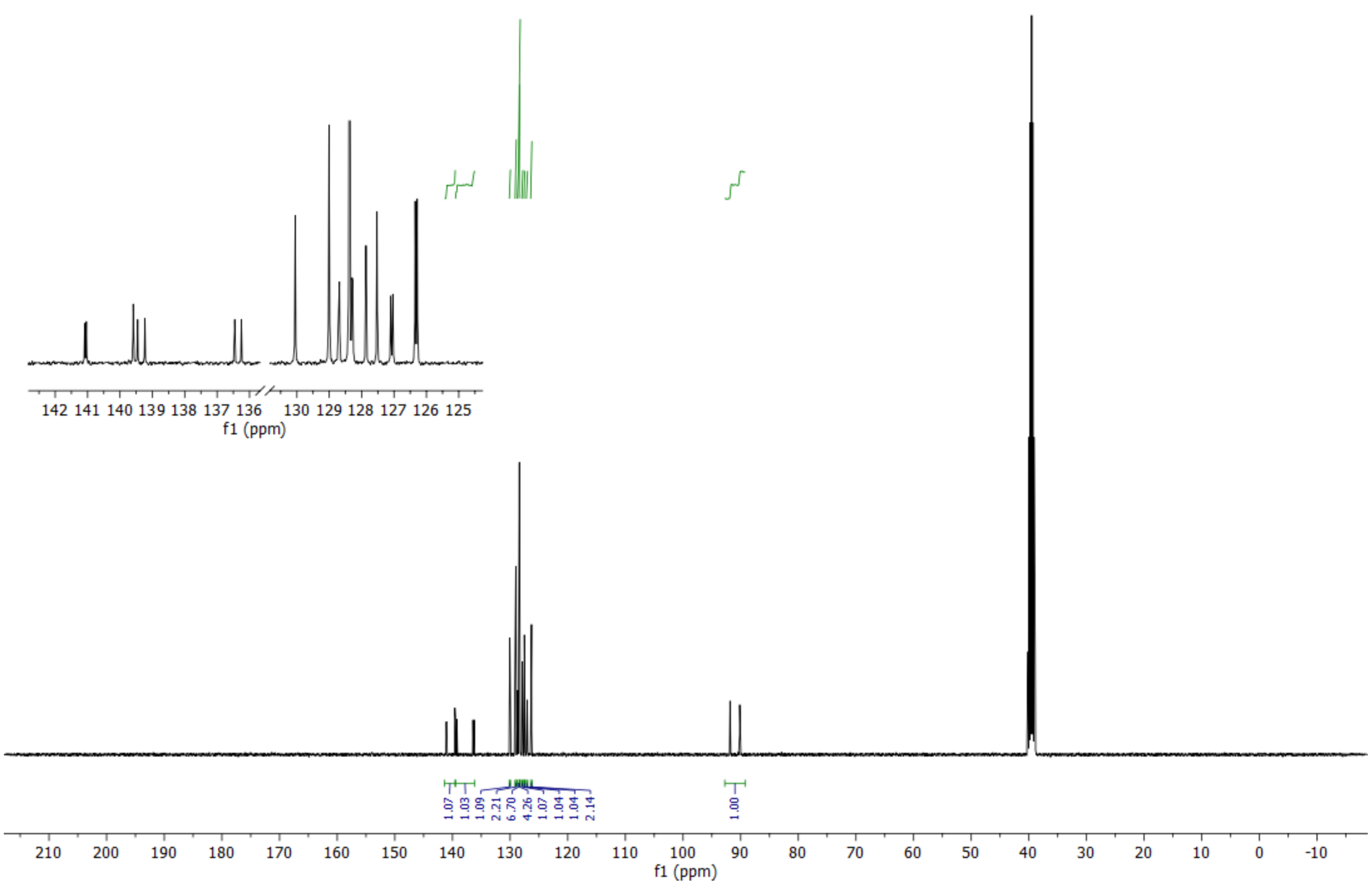

Figure S3: ^{13}C NMR Spectrum of 2-(fluorophenylmethyl)biphenyl in DMSO-*d6* measured at 101 MHz. The inset shows details of the shown signal splitting.

S2 Materials and Methods

Low-temperature scanning tunneling microscopy (STM) and X-ray photoelectron spectroscopy (XPS) were performed in two separate ultra-high vacuum (UHV) chambers with base pressures of $1 \cdot 10^{-10}$ mbar (STM) and $2 \cdot 10^{-10}$ mbar (XPS) equipped with the same facilities for sample cleaning and molecule deposition. The Ag(111) surfaces were cleaned by cycles of sputtering for 30 min (STM: Ne^{+}, $3 \cdot 10^{-5}$ mbar, 1 μA, 1.3 keV; XPS: Ar^{+}, $3 \cdot 10^{-6}$ mbar, 4 μA, 1.3 keV) and annealing for 20 min (STM: 870 K; XPS: 850 K to 900 K).

2-(Fluorophenylmethyl)biphenyl was filled into glass crucibles that are attached via angle valves to separately pumped UHV chambers, called molecule deposition chambers. Pressures of $1 \cdot 10^{-7}$ mbar (STM) and $1 \cdot 10^{-6}$ mbar (XPS) within the molecule deposition chambers were adjusted via the angle valves from the room temperature vapor pressures. The samples in the preparation chambers were exposed through gate valves to the expanded reactant gas. The deposition times were 5 s (STM) and 90 s (XPS) on the samples at 150 K. A pressure of $1 \cdot 10^{-6}$ mbar was installed within the molecule deposition chamber of the STM setup via a leak valve from a CO gas bottle attached to it. The gas was expanded into the preparation and the STM chamber through gate valves. Then, the STM shutter was opened for 30 s for deposition. The sample temperature stayed below 13 K during the deposition.

The samples were annealed in 25 K steps for 5 min from 150 K to 400 K on nitrogen-cooled manipulators in both chambers. CO desorbs at much lower temperature.

STM images were acquired at 5 K with the bias voltage applied to the sample. Constant current STM images were recorded by a bare Pt/Ir tip. Constant height images were acquired using a CO-functionalized tip. To achieve functionalization with a single molecule, the tip was positioned above a CO molecule with tunneling parameters for CO of 10 pA and 10 mV. The feedback loop was then switched off, and the tip was approached by 500 pm to 600 pm. Afterwards, the original tip-sample distance was restored and the feedback loop switched on. A successful transfer of the CO molecule to the tip was verified by rescanning the same surface region.

For lateral manipulation, the STM tip was positioned above a chosen part of the molecule[2] and approached from the distance set by a typical tunneling resistance of 10 GΩ for organic molecules to that set by 5 GΩ or 0.83 GΩ. At these resistances, the tip-molecule interactions are strong enough for manipulation. The STM tip was then moved across the surface along a predefined path in constant current mode. At its final position, the tip was withdrawn to the imaging distance by restoring the imaging parameters. The manipulation success was verified in a subsequent STM image.

The STM images were processed using WSxM.[3] Some STM images were processed by using a Laplace filter. The filter recalculates each STM image pixel by multiplying it with the following matrix:

$$\begin{pmatrix} 2 & 2 & 1 & 2 & 2 \\ 2 & 0 & -4 & 0 & 2 \\ 1 & -4 & -12 & -4 & 1 \\ 2 & 0 & -4 & 0 & 2 \\ 2 & 2 & 1 & 2 & 2 \end{pmatrix}$$

It yields 0 if the 24 neighboring pixels are identical, i.e., the slope equals zero. However, it enhances small changes in apparent height in eight directions (vertical, horizontal, and diagonal). It serves to display large and small apparent height changes that are difficult to visualize otherwise on the same z-scale. Note that all changes are likewise detectable in height profiles or at specific z-contrasts without Laplace filtering.

The XPS measurements were carried out at 86 K using an Mg Kα X-ray source with a power of 300 W. The binding energy scale was calibrated using the Ag 3d$_{5/2}$ peak centered at 368.2 eV. The electrons were collected by a hemispherical analyzer (PHOIBOS 100 from *Specs*) at an angle normal to the surface and detected by a multi-channeltron detector. We followed the reaction by monitoring the F 1s and C 1s regions of the XP spectra. The XPS

data was processed using CasaXPS.[4] Gaussian/Lorentzian product functions with a linear background for the F 1s spectra and a Shirley background for the C 1s spectra were used for peak fitting. The F 1s spectra were fitted with a linear background because of their sloping backgrounds.[5]

Plane-wave periodic density functional theory (DFT) computations were performed using the Vienna *Ab Initio* Simulation Package (VASP)[6] with the optPBE-vdW[7,8] density functional, which accounts for London dispersion forces through an augmented nonlocal correlation functional. The addition of London dispersion forces is necessary for studying adsorption at metallic surfaces where weak long-range interactions play a significant role.

A plane-wave basis set energy cutoff of 500 eV was used in the geometry optimizations, nudged elastic band (transition state/saddle point) computations, and single-point calculations. The projector augmented wave (PAW) method[9,10] was used to treat the core states, as implemented within VASP. A Γ-point centered $3 \times 3 \times 1$ Monkhorst-Pack[11] K-grid was used in all periodic calculations for systems adsorbed on the Ag(111) surface and in the gas phase.

All atoms were allowed to relax fully within the surface-adsorbate systems during geometry optimizations, except for the bottom two layers of the three-layer Ag(111) surfaces, which were kept fixed at the optPBE-vdW computed bulk lattice constant for Ag, resulting in Ag-Ag bond lengths of 0.295 nm. A z-direction dipole correction perpendicular to the Ag(111) surface was applied.

Single-point calculations on optimized structures were performed to obtain work functions (Φ), Bader charges (q), charge densities (ρ), and charge density difference (CDD, $\Delta\rho$) isosurfaces.

The work function was computed as 4.71 eV using the 1.772 nm $\times$ 1.535 nm $\times$ 3.000 nm unit cell, in good agreement with the experimental value of (4.72 ± 0.02) eV.[12] The CDD isosurfaces were calculated by subtracting the charge density of the clean Ag(111) surface and that of each isolated adsorbate species from the total surface-adsorbate system charge

density. The red and blue CDD isosurfaces indicate charge gain and loss, respectively.

The unit cell dimensions used for all monomer calculations were 1.772 nm $\times$ 1.535 nm $\times$ 3.000 nm, with all three axes chosen being large enough to ensure sufficient vacuum space and prevent unwanted periodic interactions in the z-direction and between adsorbed structures in the x- and y-directions. The unit cell dimensions were 2.362 nm $\times$ 2.046 nm $\times$ 3.000 nm for the dimer models, 2.953 nm $\times$ 2.558 nm $\times$ 3.000 nm for the tetramer models, and 3.544 nm $\times$ 3.581 nm $\times$ 3.000 nm for the trimer and hexamer models.

Adsorption energies (E_{ads}) and binding energies (E_{BE}) were computed for the surface-adsorbate structures with the following equations:

$$E_{\text{ads}} = E_{\text{surface+adsorbate}} - E_{\text{gas phase}} - E_{\text{surface}} \tag{1}$$

$$E_{\text{b}} = E_{\text{surface+adsorbate}} - N \times E_{\text{fluorine}} - N \times E_{\text{molecule}} - E_{\text{surface}} \tag{2}$$

where the optimized Ag(111)-adsorbate system energies ($E_{\text{surface+adsorbate}}$), optimized gas phase energies of the molecule ($E_{\text{gas phase}}$), clean optimized Ag(111) surface energies (E_{surface}), fluorine related energy (E_{fluorine}) and molecular energies (E_{molecule}) were used.

Surface-adsorbate systems that involve more than one adsorbate had their adsorption energies calculated in two different manners by modifying the usage of the $E_{\text{gas phase}}$ term: (1) treating the adsorbate molecules as a single combined entity, and (2) treating the multiple adsorbates (E_{fluorine} and E_{molecule}) as separate entities, each with their respective number N.

Local-Orbital Basis Suite Towards Electronic-Structure Reconstruction (LOBSTER)[13] calculations were performed to investigate the bonding strengths of C-F bonds, Ag-F bonds, C-Ag bonds, and C-C bonds using the integrated crystal orbital Hamilton populations (iCOHP). First-principles molecular dynamics (FPMD) movies were generated using the NVT (constant particle number N, volume V, and temperature T) ensemble at temperatures of 150 K and 325 K for the reactant molecule, 375 K for all other structures, and 450 K for the hexamer clusters. The FPMD results provide insight into the stabilities and

tendencies of the molecular species when adsorbed on the Ag(111) surface.

DFT calculations were also performed using the Gaussian[14] software package to make use of an atomic basis set code for assistance in computational structure discovery alongside the plane-wave VASP software package. The structures studied in Gaussian were generated by either importing the VASP surface-adsorbate geometry optimized structures and performing a single point calculation, or through geometry optimizations and transition state optimizations. The PBE0-D3[15,16] hybrid density functional and dispersion correction along with the Def2-TZVPP basis set were used in the Gaussian computations. The transition state optimization structures were confirmed as having one imaginary frequency with the correct reaction coordinate character of either C-F bond dissociation or C-C bond formation being detailed using vibrational frequency computations.

S3 Separation of single reactant molecules from the superstructure

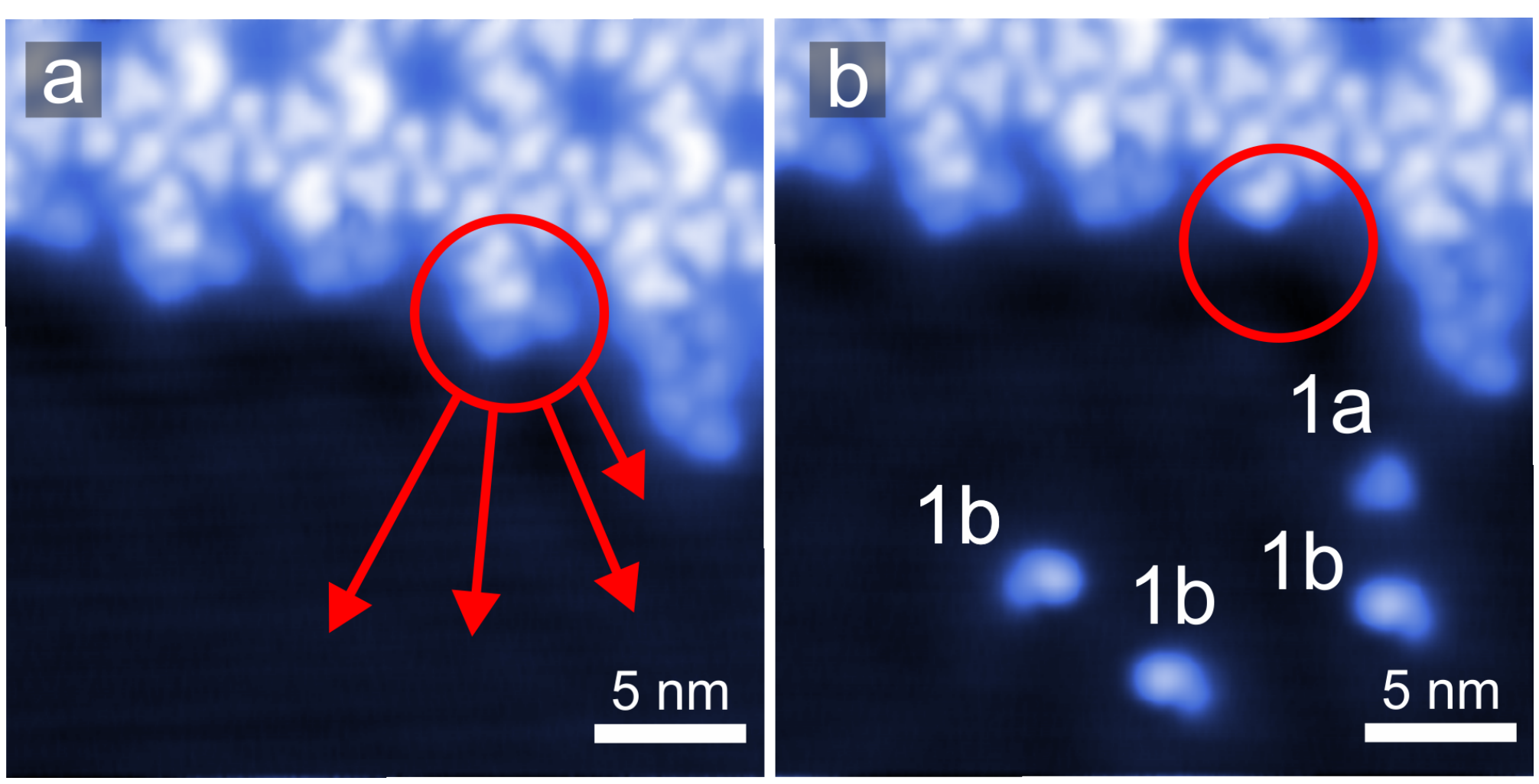

Figure S4: Separation of reactant molecules from a superstructure formed during adsorption at 150 K. (a,b) STM images before (a) and after (b) lateral manipulation along the red arrow. The manipulation resistance was 5 GΩ. The images were recorded at V = 100 mV and I = 5 pA.

The reactants form a superstructure after adsorption at 150 K. For the real space analysis of the reactants, single molecules are separated from the superstructure by lateral manipulation (Fig. S4). Two different molecules are separated from the superstructure. The first one, marked as **1a**, is identified in the main text as the conformer with the F atom pointing toward the vacuum and phenyl ring III is parallel to the surface.

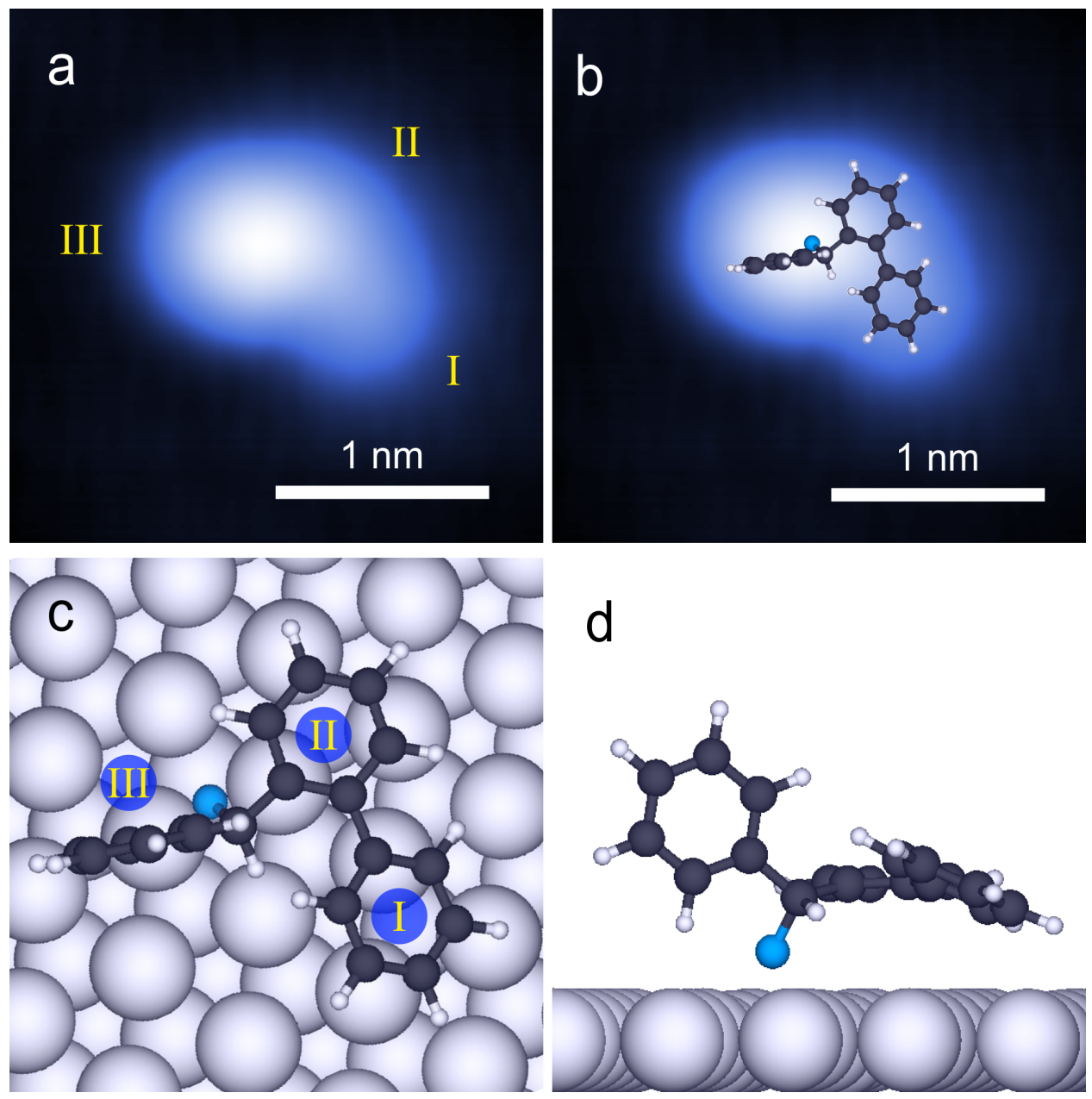

Figure S5: Structural characterization of the reactant **1b**. (a) Small scale image after manipulation from the superstructure formed at 150 K. (b) Superposition of the molecule model on the real-space structure. (c,d) Top view (c) and side view (d) of the calculated geometries of the reactant 1b. The image was recorded at V = 100 mV and I = 5 pA.

The second molecule **1b** is imaged as a single, curved bright protrusion in the STM (Fig. S5a). To correlate this STM characteristic with the molecular structure, an optimized structure of the reactant on Ag(111) is calculated with DFT (Fig. S5c,d). In the optimized structure, phenyl ring III points away from the surface, phenyl rings I and II are parallel to the surface, and the F atom points toward the surface. Accordingly, the bright protrusion is assigned to phenyl ring III, while phenyl rings I and II are imaged with a lower apparent height. The DFT optimized structure reproduces the geometry observed in the STM image well (Fig. S5b).

S4 Superposition of molecule models on real-space structures

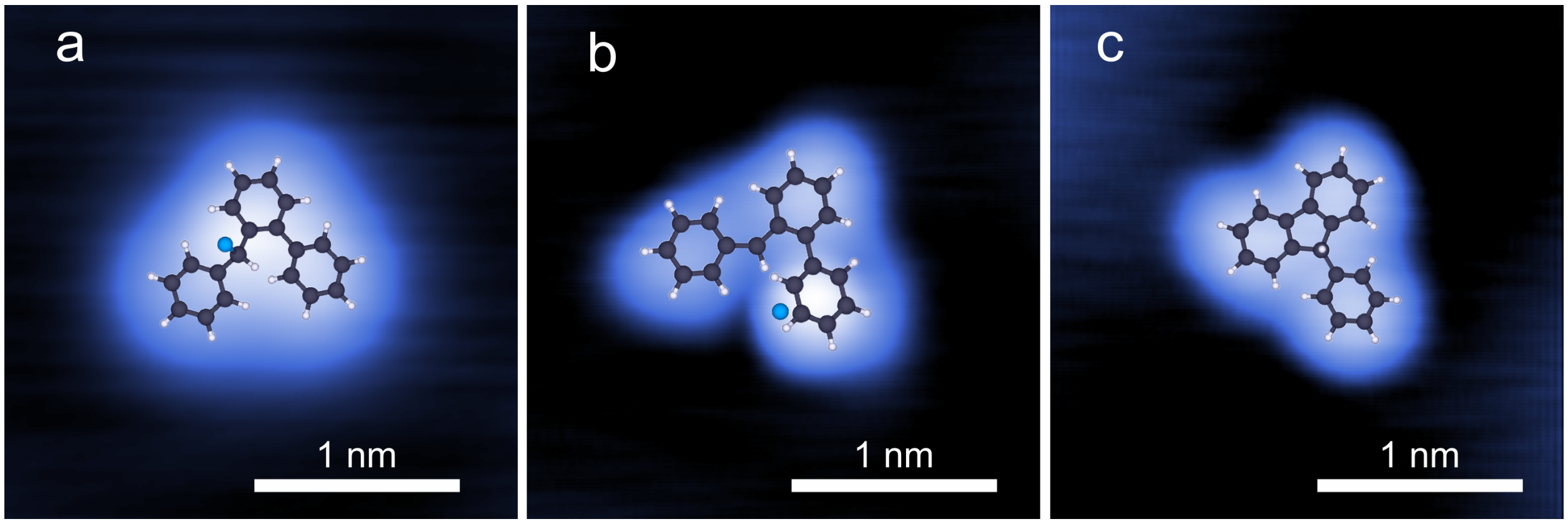

Figure S6: Superposition of molecule models on real-space structures. Superimposed molecule models of reactant **1a** (a), intermediate **2b** (b), and product **3b** (c) with STM images to scale. The images (a,b,c) were recorded at 50 mV, 10 pA.

In the main text, the STM images and the DFT calculated structures of the reactant, intermediate complex, and product are described in detail. Here, the DFT-calculated structures are superimposed to the respective STM images (Fig. S6). The DFT calculated structures nicely reflect the symmetry and size of the protrusions in the STM images.

S5 Superstructure characterization

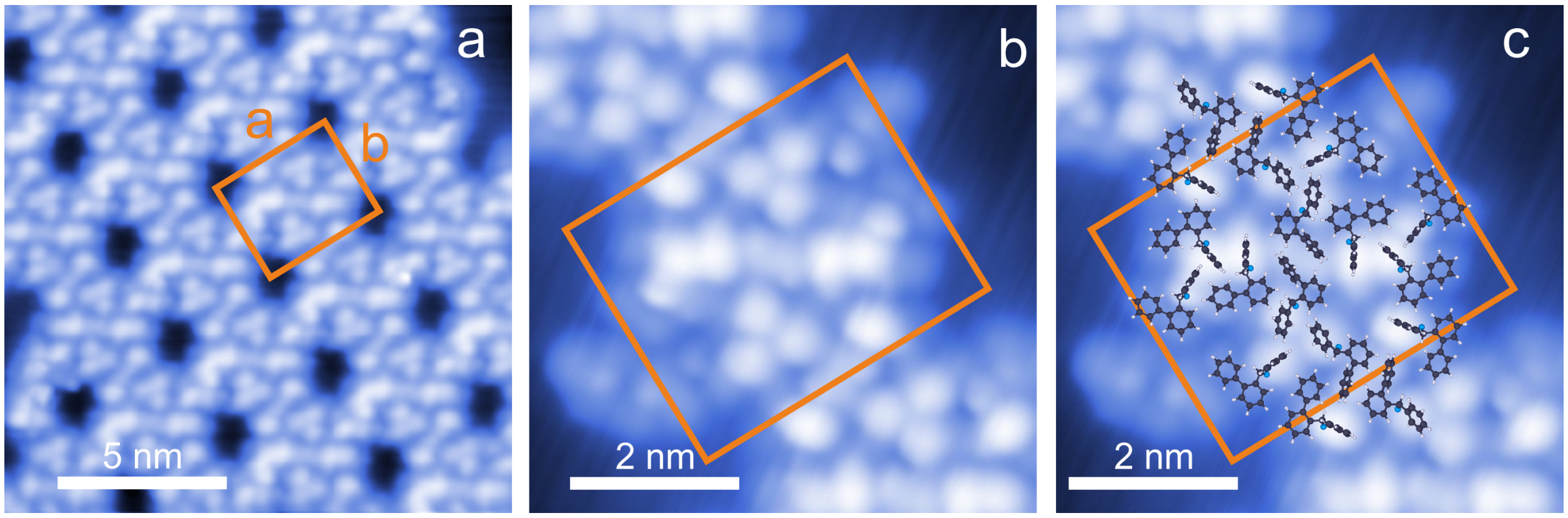

Figure S7: Superstructure characterization: overview image of the superstructure (a) and high-resolution STM images recorded with a CO tip (b,c). The unit cell is marked by the orange rectangles. Panels (b,c) are rotated to match the orientation of the unit cell in (a). (c) Superimposed molecular models of reactant **1b**. The images were recorded at (a) 50 mV, 5 pA and (b,c) 100 mV, 10 pA.

The reactants form a superstructure after adsorption at 150 K, as described in the main text. Here, we analyze this superstructure further. It has a rectangular unit cell with $a = 3.77$ nm and $b = 3.10$ nm (Fig. S7a). Its internal structure is better resolved in the high-resolution images recorded with a CO-functionalized tip (Fig. S7b). The bright protrusions are attributed to the phenyl rings pointing toward the vacuum, as in reactant **1b**. On this basis, we propose a molecular model (Fig. S7c), in which the unit cell contains 14 reactants. In this model, some reactants have their phenyl ring I rotated relative to the parallel orientation in **1b** to reproduce the contrast in the STM. From the resulting relative orientations, we attribute the stabilizing interactions to $\pi\cdots\pi$, C-H$\cdots\pi$ and C-H$\cdots$F-C interactions. We emphasize that this model represents one plausible arrangement consistent with the STM data. Alternative orientations and arrangements of the molecules within the unit cell cannot be excluded. A definitive assignment would require DFT relaxation of the full 14-molecule superstructure including dispersion interactions and the metal substrate, which is computationally prohibitive. We therefore restrict the DFT calculations to the single adsorbed molecule, which is the structurally relevant unit for the intramolecular coupling

reaction discussed in the main text.

S6 Apparent height profiles of the reactant, intermediate, and product

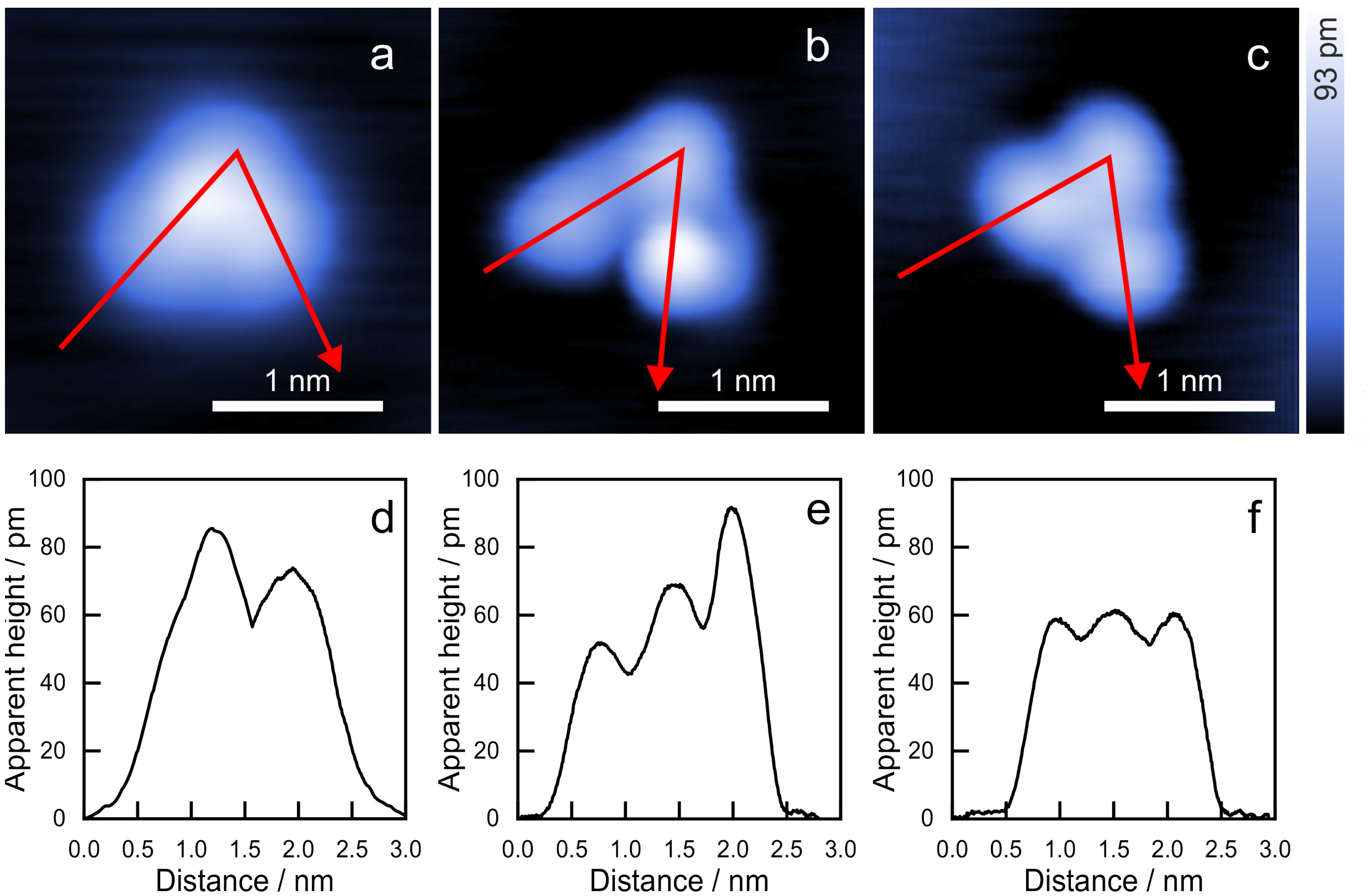

Figure S8: Apparent height profile of the reactant **1a**, intermediate **2b**, and product **3b**. (a,b,c) STM images of (a) the reactant, (b) the intermediate, and (c) the product with the height profiles (d,e,f) of (d) the reactant, (e) the intermediate, and (f) the product along the arrows in (a,b,c). The images were recorded at V = 50 mV, I = 10 pA.

In the main text, the heights of the reactant, intermediate complex, and product are discussed. Here, we present the apparent height profiles (Fig. S8). The height profile of the reactant is characterized by two prominent peaks (Fig. S8d). The first peak, with an apparent height of 86 pm, is attributed to the fluorine atom pointing toward the vacuum. The second peak, at 74 pm, is assigned to phenyl ring I. The remaining two phenyl rings appear

as a shoulder in the profile.

In contrast, the height profile of the intermediate complex exhibits three distinct maxima at 52 pm, 69 pm, and 92 pm, which are assigned to the three phenyl rings (Fig. S8e). The highest peak is attributed to a tilted phenyl ring that interacts with the underlying fluorine atom, as discussed in the main text.

The height profile of the product molecule also shows three peaks corresponding to the three phenyl rings (Fig. S8e). These peaks appear at similar heights ($\approx$ 61 pm), reflecting the relatively planar geometry of the product molecule.

S7 Tetramers of the product

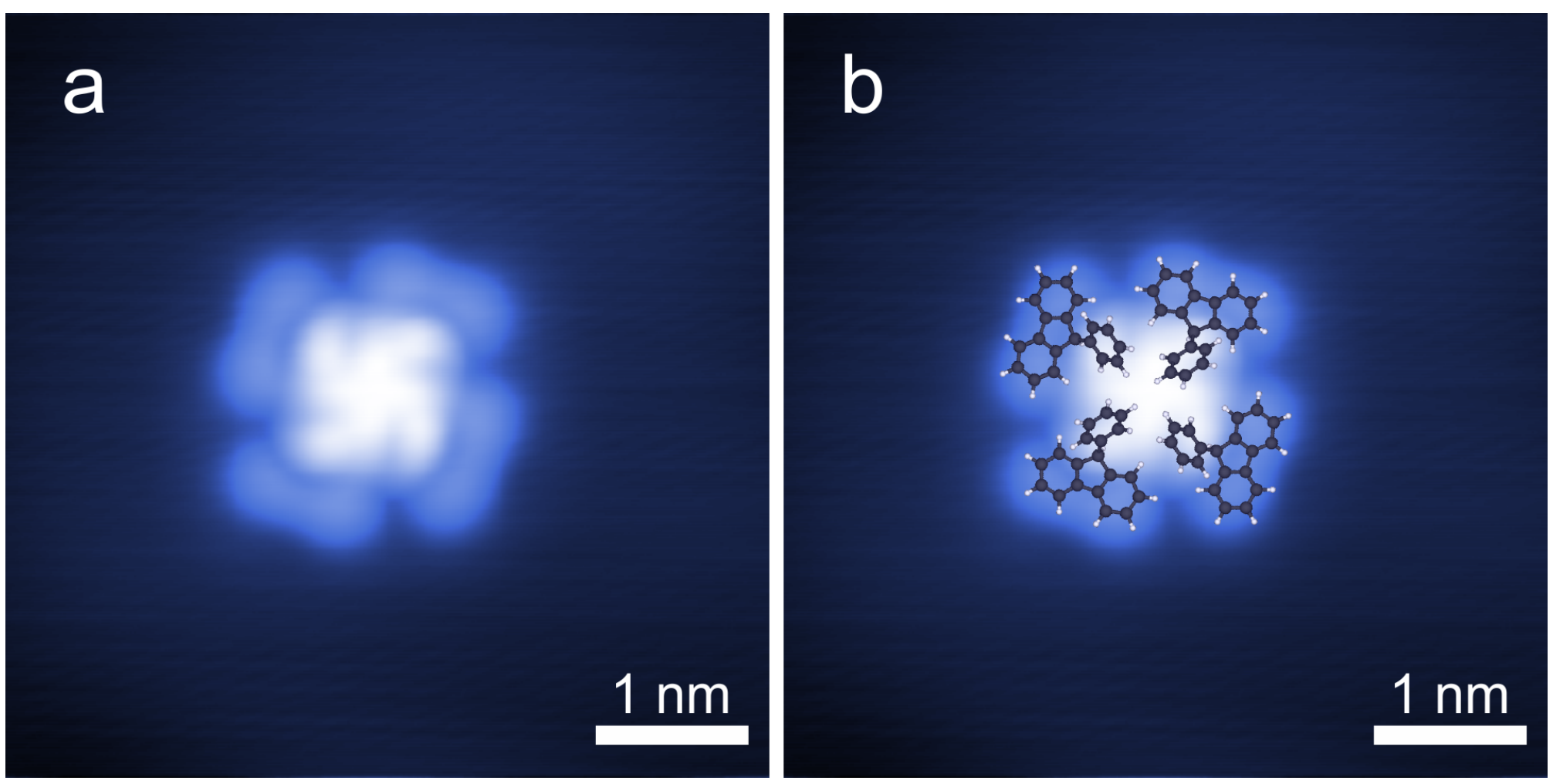

Figure S9: Tetramer of products. (a) STM image and (b) superimposed molecule models with STM image to scale. V = 50 mV and I = 500 pA.

In the main text, we discussed the predominantly isolated product. It adopts a configuration with all phenyl rings parallel to the surface and the H atom bonded to the Csp^3 atom pointing toward the vacuum. In addition to these isolated product species, a small number of clusters are observed after annealing at 400 K. These clusters have a square shape (Fig. S9a). The core of this cluster is of higher apparent height than its border. The surrounding four peanut-shaped protrusions of lower apparent height are on the four sides of the square. The size

of the peanut-shaped protrusions suggests that they represent two phenyl rings, assigned here to the fluorene moiety of the product molecule. The higher apparent height of the core suggests that the phenyl ring III of the product is pointing away from the surface.

In the gas phase the product adopts a non-planar geometry. In this geometry, the single phenyl ring orients perpendicular to the fluorene moiety. The geometry with the phenyl ring pointing toward the vacuum is adopted in the tetramers. In this structure, the phenyl rings of the product are slightly rotated from the single product structure to better interact with each other via a $\pi \cdots \pi$ interaction (Fig. S9b).

S8 Computational results

S8.1 Gas-phase structure of the reactant

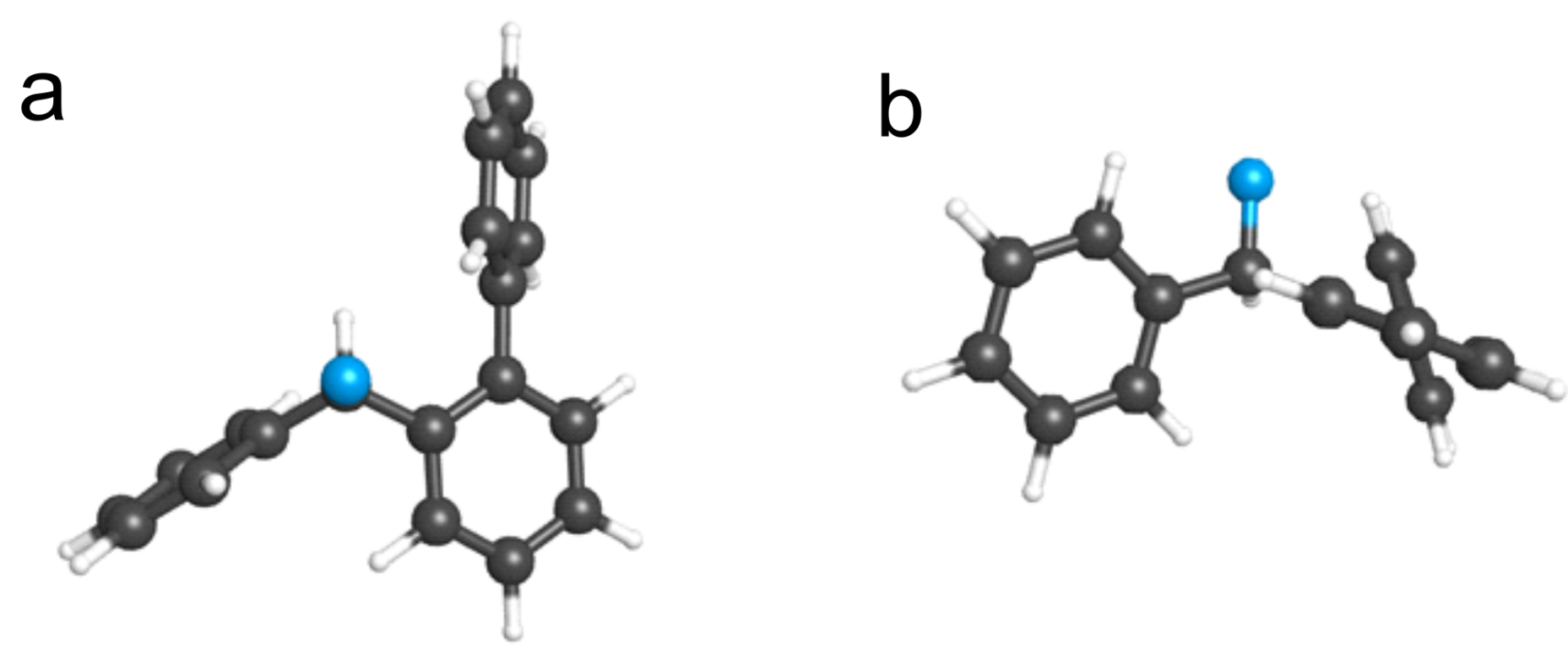

Figure S10: (a) Top view and (b) side view of the gas-phase geometry optimized structure of the reactant.

In the main text, we discuss the structure of the reactant on the Ag(111) surface. In the gas phase, the reactant adopts a geometry in which its phenyl rings are rotated relative to each other (Fig. S10). This geometry minimizes steric repulsion between the hydrogen atoms of the phenyl rings.

S8.2 FPMD movies

Our first-principles molecular dynamics (FPMD) simulations on the intermediate complex using a NVT ensemble at 375 K over 15 ps show that the molecule diffuses along the surface with the fluorine atom following close by even though it is chemisorbed to the Ag(111) surface and capable of finding its own preferred binding site. This observation shows that even at mildly elevated temperatures, the intermediate complex diffuses well and the fluorine and carbon-based intermediate are most stable when the fluorine is inserted nearby. Further, it is difficult to fully break the Ag-C interaction in the intermediate. This FPMD result is supported by the binding energy of the intermediate complex being at -6.19 eV. The FPMD movie clearly shows that the fluorine follows the intermediate and that both species can easily diffuse along the surface through bridge sites. Lastly, our FPMD movie on the intermediate monomer showing that C-C coupling does not occur at 375 K suggests that the reaction requires a longer timescale to break the Ag-C interaction or needs further assistance through intermolecular interactions and removal of the fluorine to reform the positively charged sp^2 carbon. In fact, our FPMD movies over $\sim$1 ps with the intermediate hexamer at 375 K and 450 K reveal that intermolecular interactions may lead to the Ag-C interaction breaking to then facilitate C-C coupling to occur. With the dimer intermediate at 375 K, the fluorines still follow the intermediate molecules, but the fluorines remain in close proximity to each other while allowing one molecule to spin away from a fluorine atom which may expose it to C-C coupling. See the supporting information for the FPMD movies.

The FPMD movies of the reactant molecule show that the molecule is capable of staying intact without C-F dissociation at 150 K and 325 K when starting in either the fluorine up or the fluorine down structures. However, the result at 325 K of the molecule remaining intact is likely a byproduct of the FPMD simulation being incapable of bypassing the transition state for C-F bond dissociation since FPMD locates minima instead of maxima on the potential energy surface. The FPMD movies also show that the reactants remain adsorbed to the surface at these temperatures and freely convert between the fluorine up and the fluorine

down structures. This observation supports the rationale that the fluorine is capable of pointing down toward the Ag(111) surface from its thermodynamically more stable structure with the fluorine pointing up and properly interact the fluorine with a reactive surface site in order for the intermediate complex to form.

An FPMD movie at 375 K of a destabilized intermediate complex forms the product through C-C coupling, which shows that when the molecule is destabilized from the intermediate complex, it will react and form the product and a hydrofluoric acid molecule. When the central carbon's hydrogen is destabilized, the product also forms through C-C coupling and a coinciding hydride shift occurs to reform aromaticity and place the hydrogen on the central carbon as expected in the product. Of note, when the intermediate structure is put into its C-C coupled structure prior to deprotonation, the structure is capable of falling back into the intermediate chemisorbed species instead of deprotonating into the product. This further supports the high stability of the intermediate complex when bonding in close proximity with the Ag(111) surface. Lastly, the phenyl rings are capable of freely rotating at as low as 150 K temperatures so long as a fluorine atom is not underneath a ring to prevent this as in the intermediate complex.

S8.3 Charge Density Difference (CDD) isosurfaces

Charge density difference (CDD) isosurfaces reveal the modulation of charge density that occurs from adsorption to the Ag(111) surface from the structures discussed in the main text.

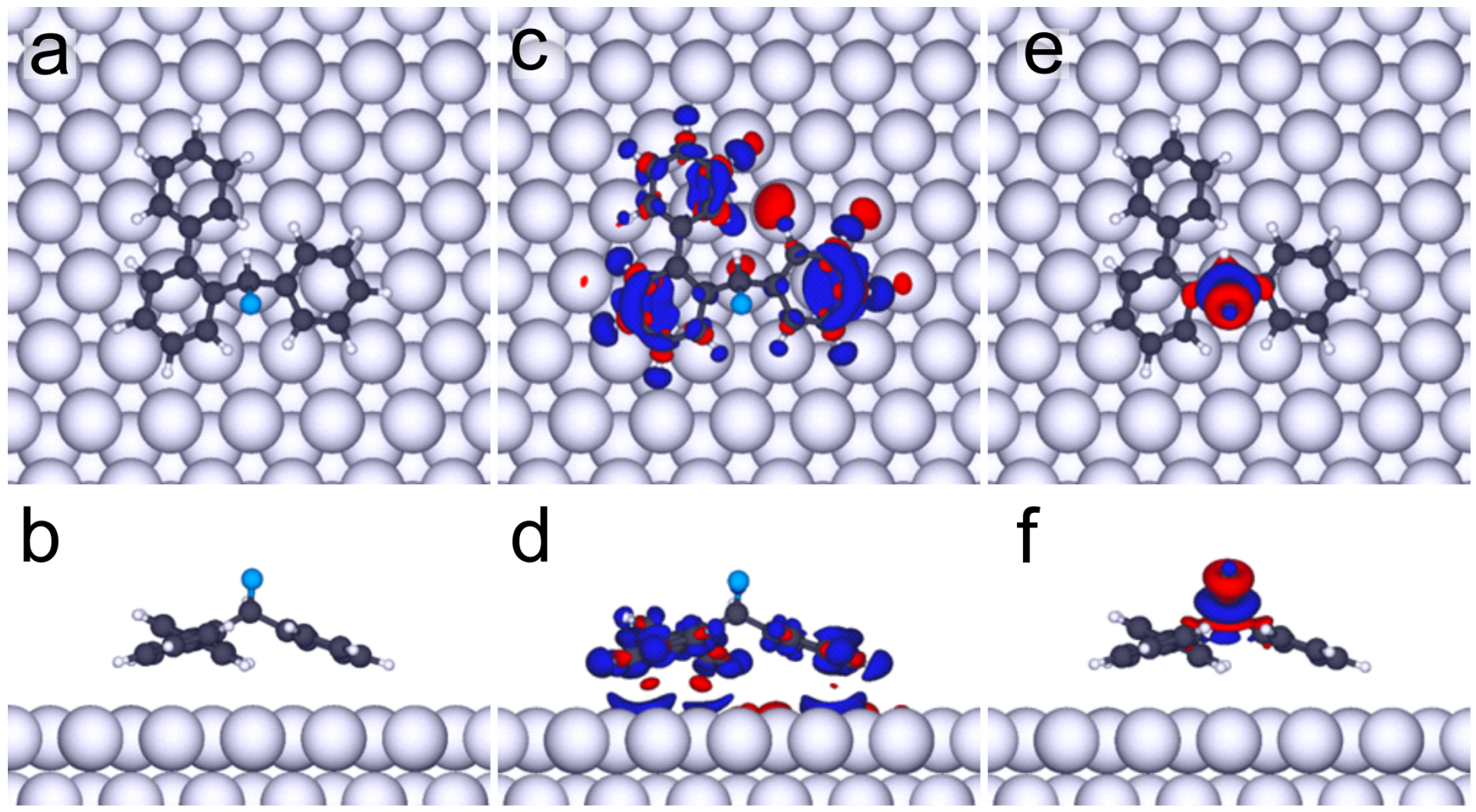

Figure S11: (a,c,e) Top views and (b,d,f) side views of the calculated geometry (a,b) and the CDD isosurfaces (c to f) of reactant **1a**. (c,d) CDD while treating the fluorine as part of the charge density of the adsorbate. (e,f) CDD while treating the fluorine as a separate component of the charge density of the adsorbate. The isovalues are (c,d) ±0.003 e/Å^3 and (e,f) ±0.03 e/Å^3.

For the reactant **1a** in the structure with the fluorine pointing up, the CDD charge gain and loss from adsorption are primarily through the rings and does not involve the fluorine atom (Fig. S11).

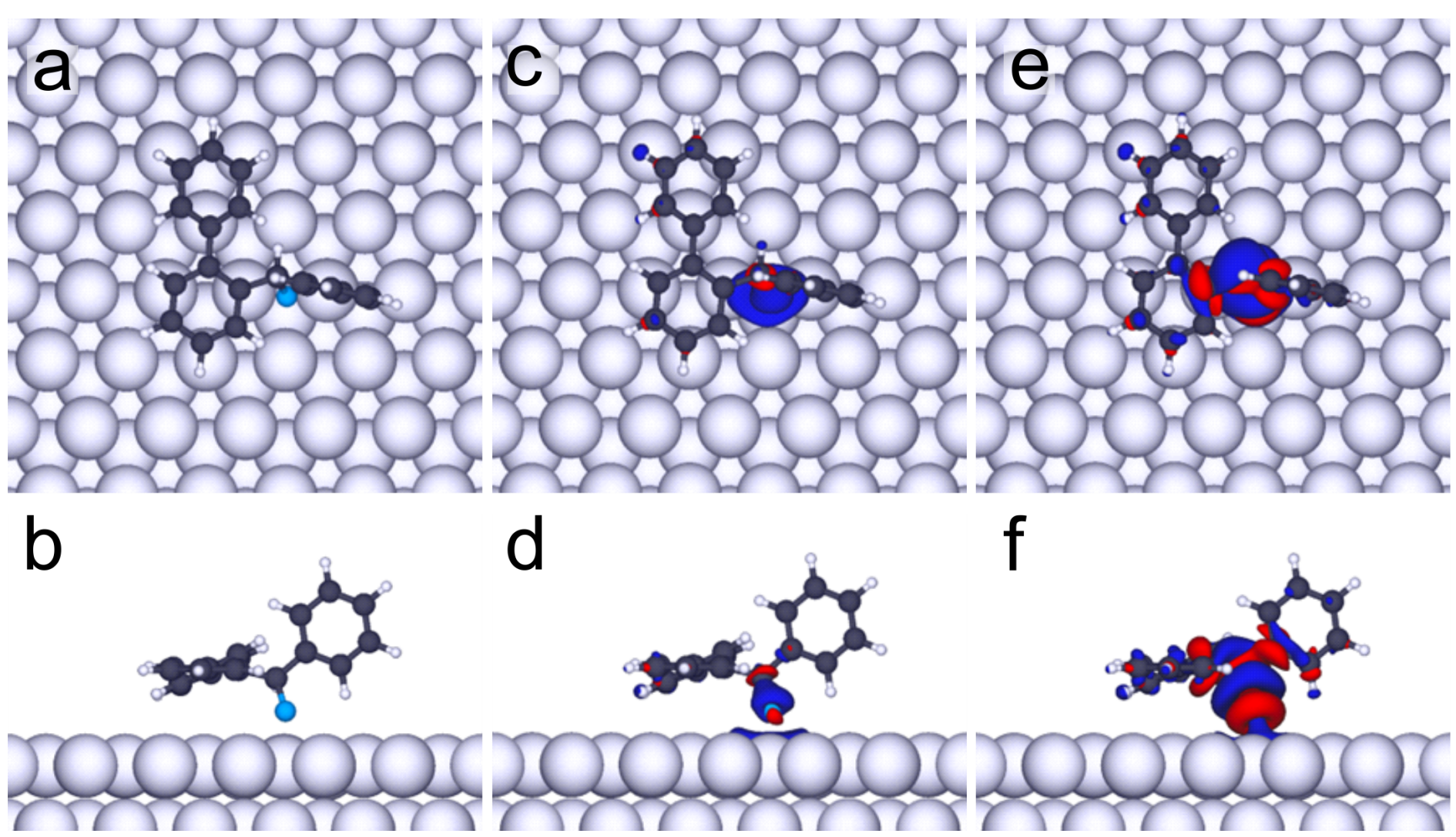

Figure S12: (a,c,e) Top views and (b,d,f) side views of the calculated geometry (a,b) and the CDD isosurfaces (c to f) of reactant **1b**. (c,d) CDD while treating the fluorine as part of the charge density of the adsorbate. (e,f) CDD while treating the fluorine as a separate component of the charge density of the adsorbate. The isovalues are ±0.009 e/Å^3.

The CDD isosurfaces are very different when the fluorine atom is pointing down toward the surface in reactant **1b** (Fig. S12). Here, the fluorine atom is gaining a significant amount of charge density from the Ag(111) surface, and the C-F bond loses charge which displays the capability of the Ag(111) surface to lower the transition barrier to C-F bond dissociation and produce chemisorbed fluorine.

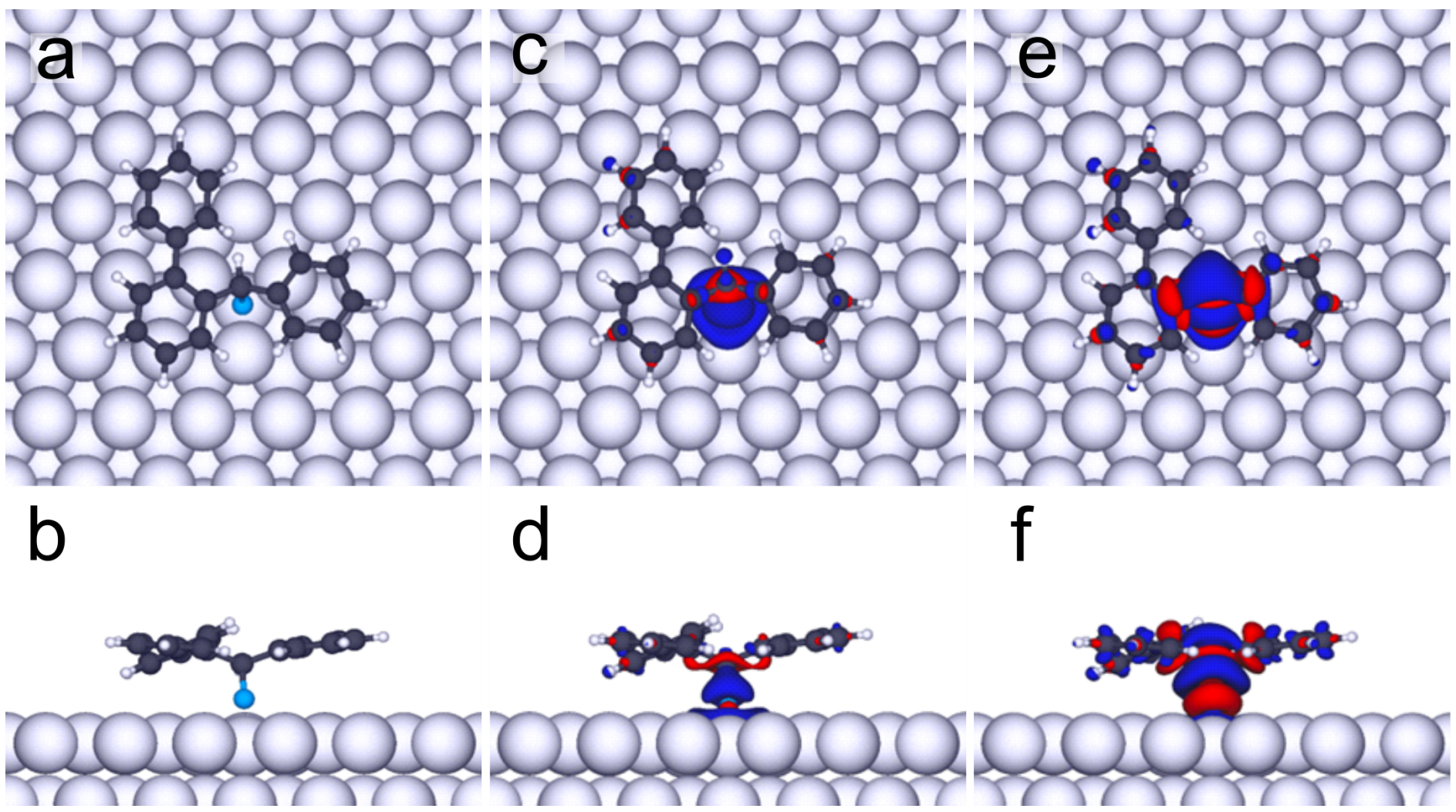

Figure S13: (a,c,e) Top views and (b,d,f) side views of the calculated geometry (a,b) and the CDD isosurfaces (c to f) of reactant **1c**. (c,d) CDD while treating the fluorine as part of the charge density of the adsorbate. (e,f) CDD while treating the fluorine as a separate component of the charge density of the adsorbate. The isovalues are ± 0.009 e/Å^3 (c to f).

The CDD of the reactant **1c** displays the same interaction between the fluorine and the Ag(111) surface as reactant **1b** (Fig. S13), but the charge modulation is more pronounced here with reactant **1c**.

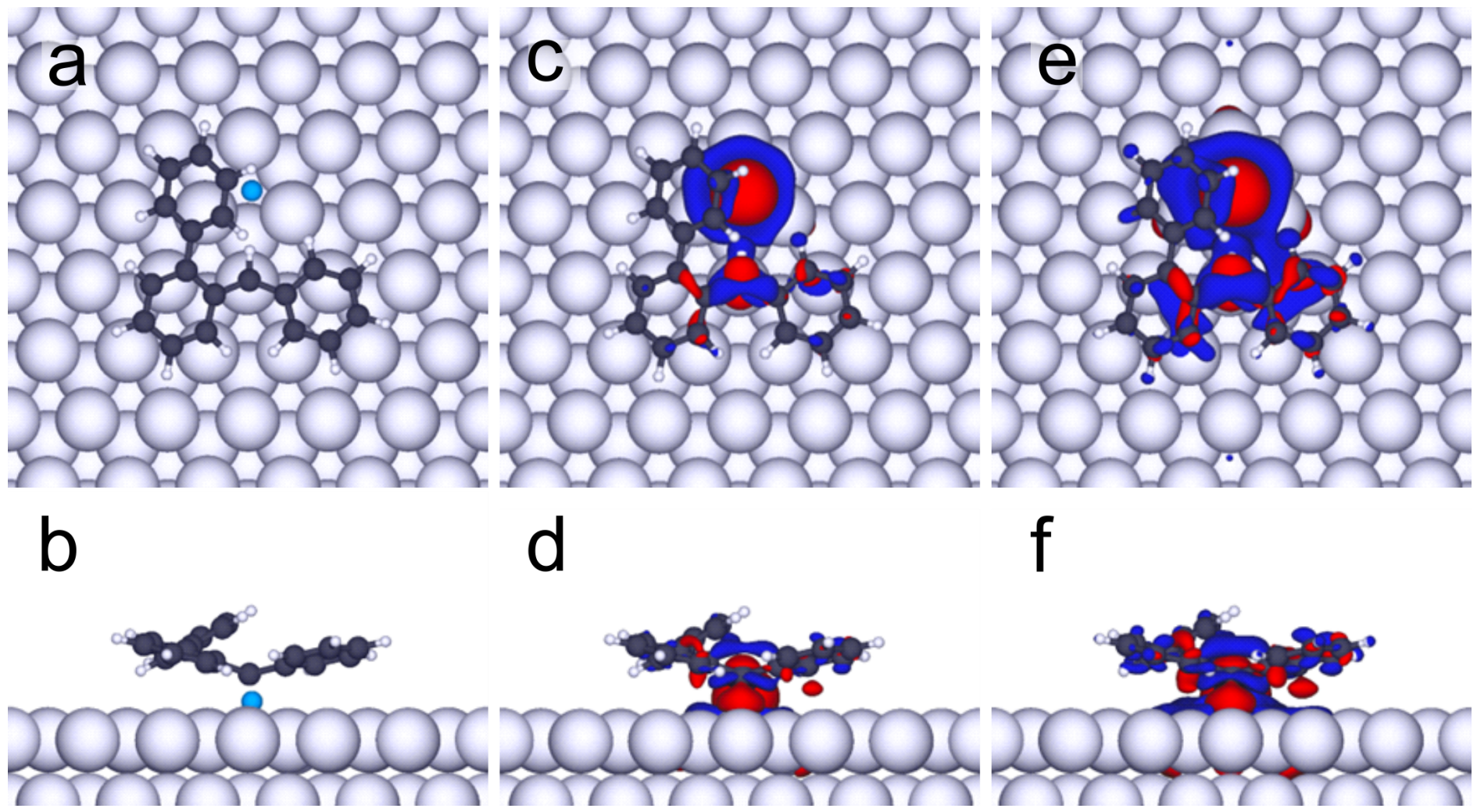

Figure S14: (a,c,e) Top views and (b,d,f) side views of the calculated geometry (a,b) and the CDD isosurfaces (c to f) of intermediate complex **2b**. (c-f) CDD while treating the fluorine as a separate component of the charge density of the adsorbate. The isovalues are ± 0.009 e/Å^3 (c,d) and ± 0.006 e/Å^3 (e,f).

The CDD for the intermediate complex **2b** shows a significant amount of charge gain directly between the Ag(111) surface and the central carbon (Fig. S14). This charge gain is detailing Ag-C interaction and supports the central carbon as being negatively charged when it was previously positively charged after C-F bond dissociation. The fluorine atom has a large amount of charge gain on it and a charge depression created around it on the Ag(111) surface similar to what is seen with chlorine chemisorption on Ag(111).[17] This fluorine atom, when underneath the biphenyl ring, pulls charge density out of the ring, making it less negatively charged and thus less reactive (deactivated) for Friedel-Crafts alkylation purposes.

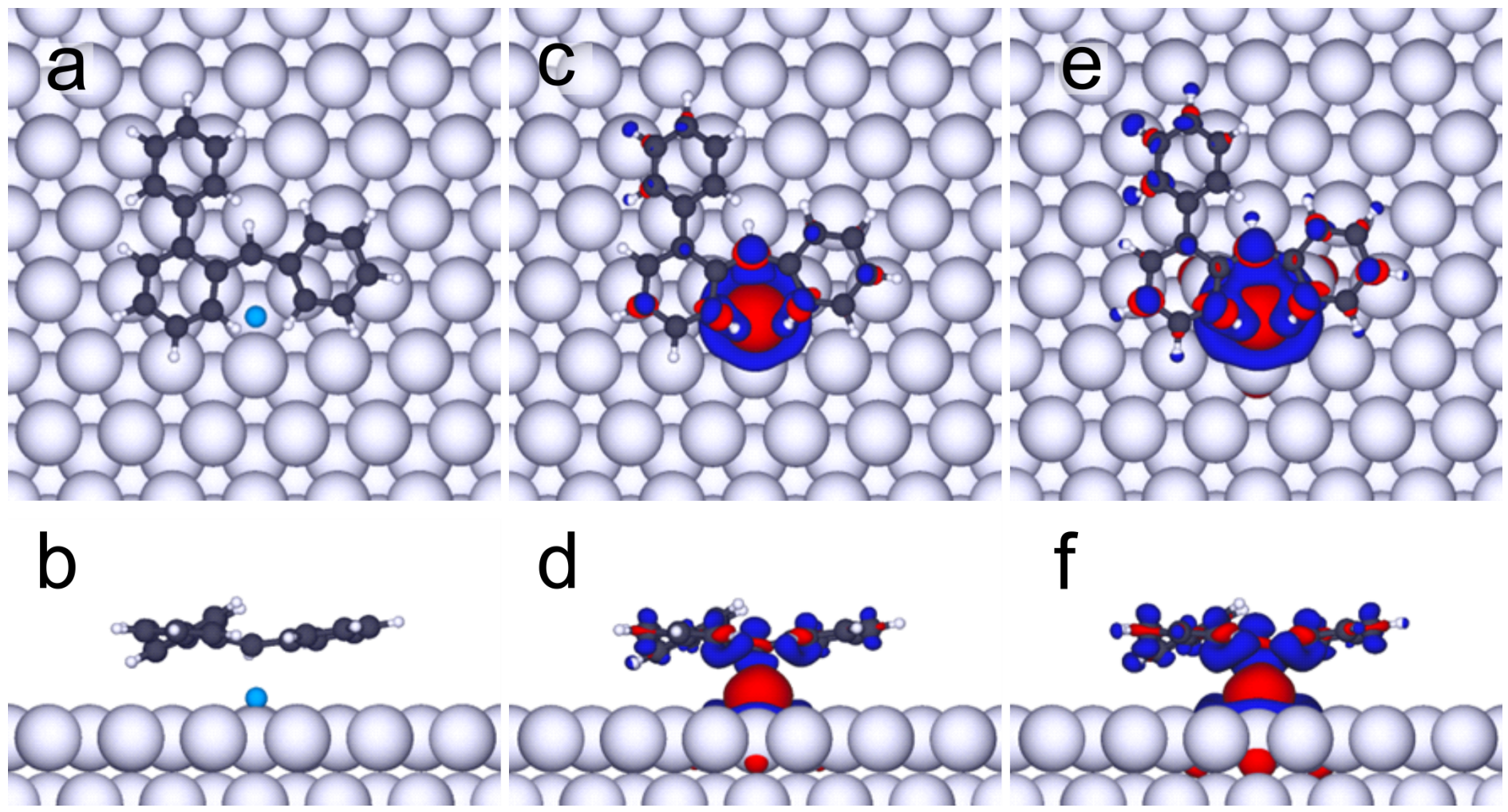

Figure S15: (a,c,e) Top views and (b,d,f) side views of the calculated geometry (a,b) and the CDD isosurfaces (c to f) of intermediate complex **2a**. (c-f) CDD while treating the fluorine as a separate component of the charge density of the adsorbate. The isovalues are ± 0.009 e/Å^3 (c,d) and ± 0.006 e/Å^3 (e,f).

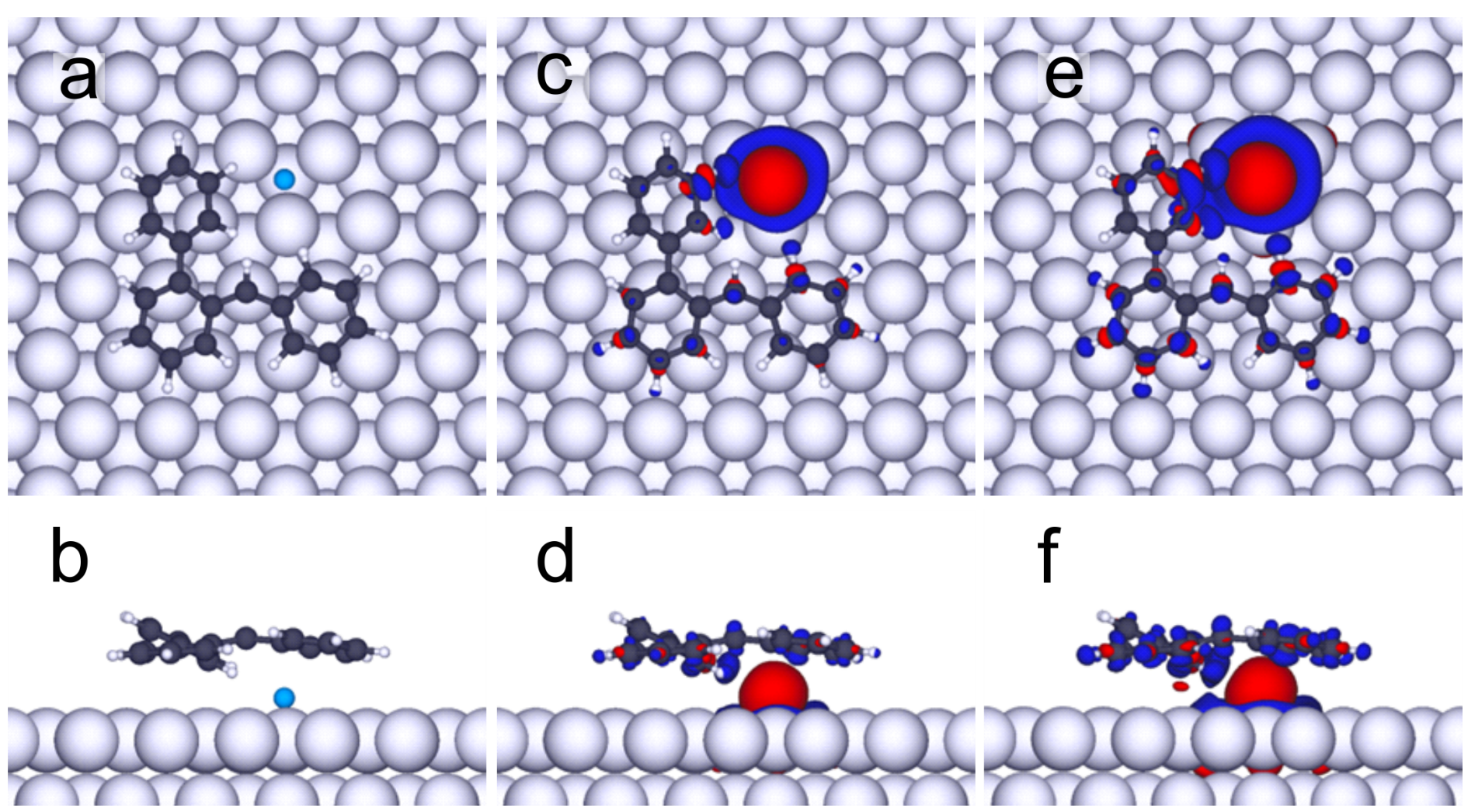

Figure S16: (a,c,e) Top views and (b,d,f) side views of the calculated geometry (a,b) and the CDD isosurfaces (c to f) of intermediate complex **2c**. (c-f) CDD while treating the fluorine as a separate component of the charge density of the adsorbate. The isovalues are ± 0.009 e/Å^3 (c,d) and ± 0.006 e/Å^3 (e,f).

The CDD isosurfaces of the intermediate complex immediately after C-F bond dissociation **2a** (Fig. S15) or after breaking the Ag-C interaction **2c** (Fig. S16) reveal that the central carbon is no longer gaining charge from the Ag(111) surface. Therefore, breaking the Ag-C interaction returns electrons to the Ag(111) surface and makes the molecule positively

charged and ready for Friedel-Crafts alkylation.

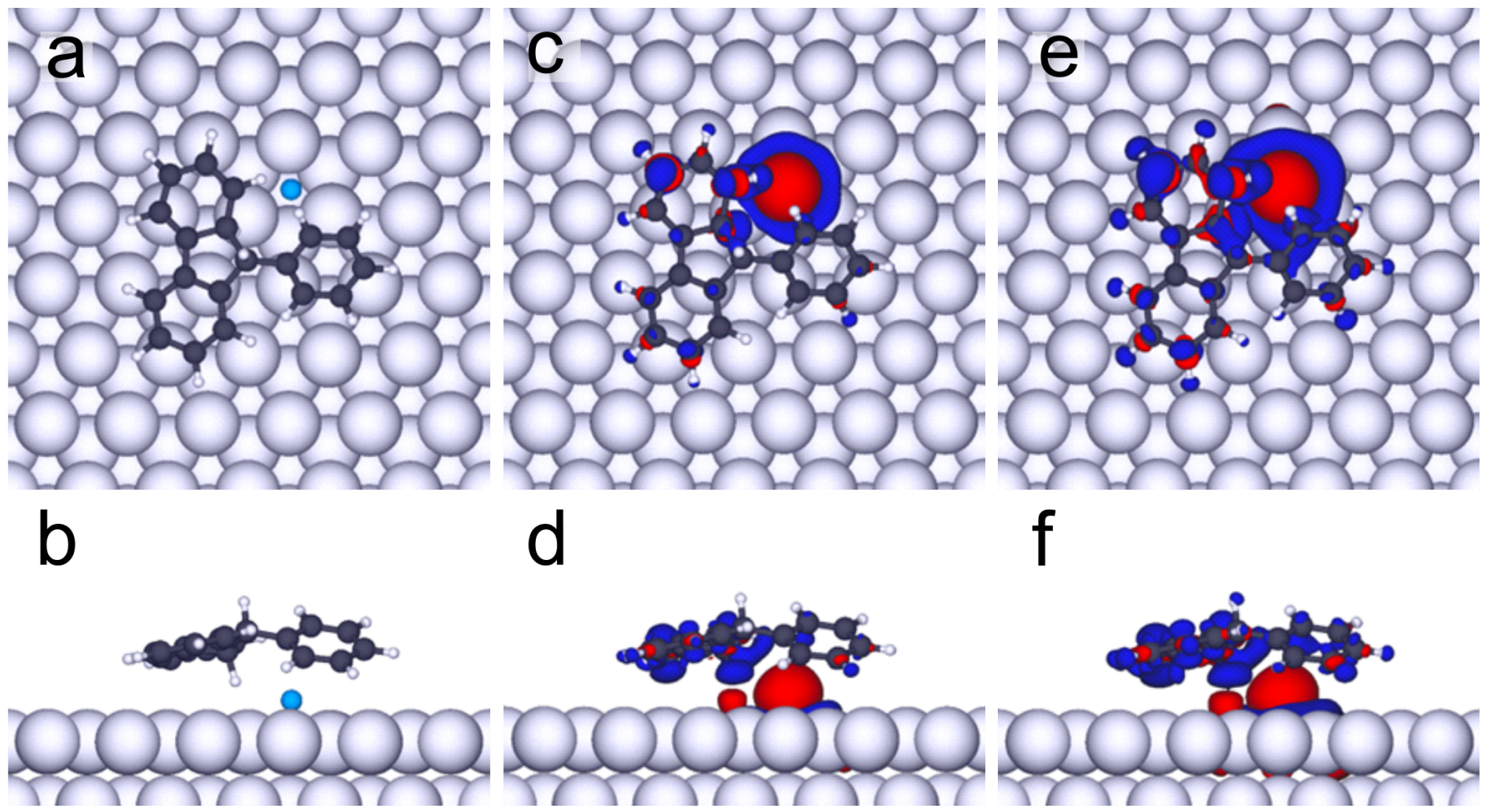

Figure S17: (a,c,e) Top views and (b,d,f) side views of the calculated geometry (a,b) and the CDD isosurfaces (c to f) of the C-C coupled structure **3-H**. (c-f) CDD while treating the fluorine as a separate component of the charge density of the adsorbate. The isovalues are ± 0.009 e/Å^3 (c,d) and ± 0.006 e/Å^3 (e,f).

The CDD isosurfaces of the C-C coupled structure **3-H** reveal that the surface and the reactive proton have charge gain between the two, which supports the ability for the proton to transfer directly to the Ag(111) surface and form Ag-H bonding (Fig. S17). The hydrogen would then diffuse to the fluorine atom to form and subsequently desorb HF molecules.

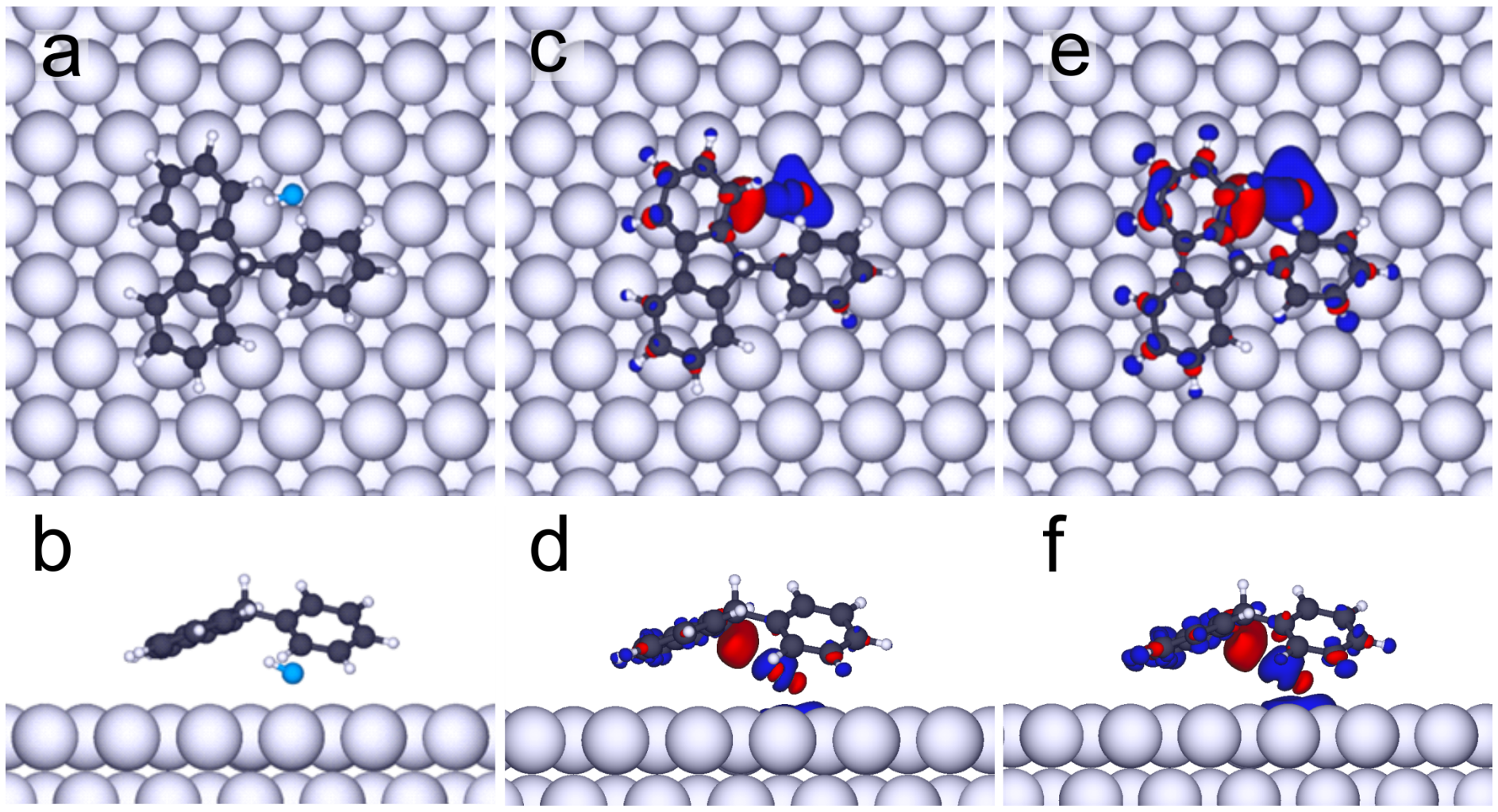

Figure S18: (a,c,e) Top views and (b,d,f) side views of the calculated geometry (a,b) and the CDD isosurfaces (c to f) of product **3a**. (c-f) CDD while treating the HF molecule as a separate component of the charge density of the adsorbate. The isovalues are ± 0.009 e/Å^3 (c,d) and ± 0.006 e/Å^3 (e,f).

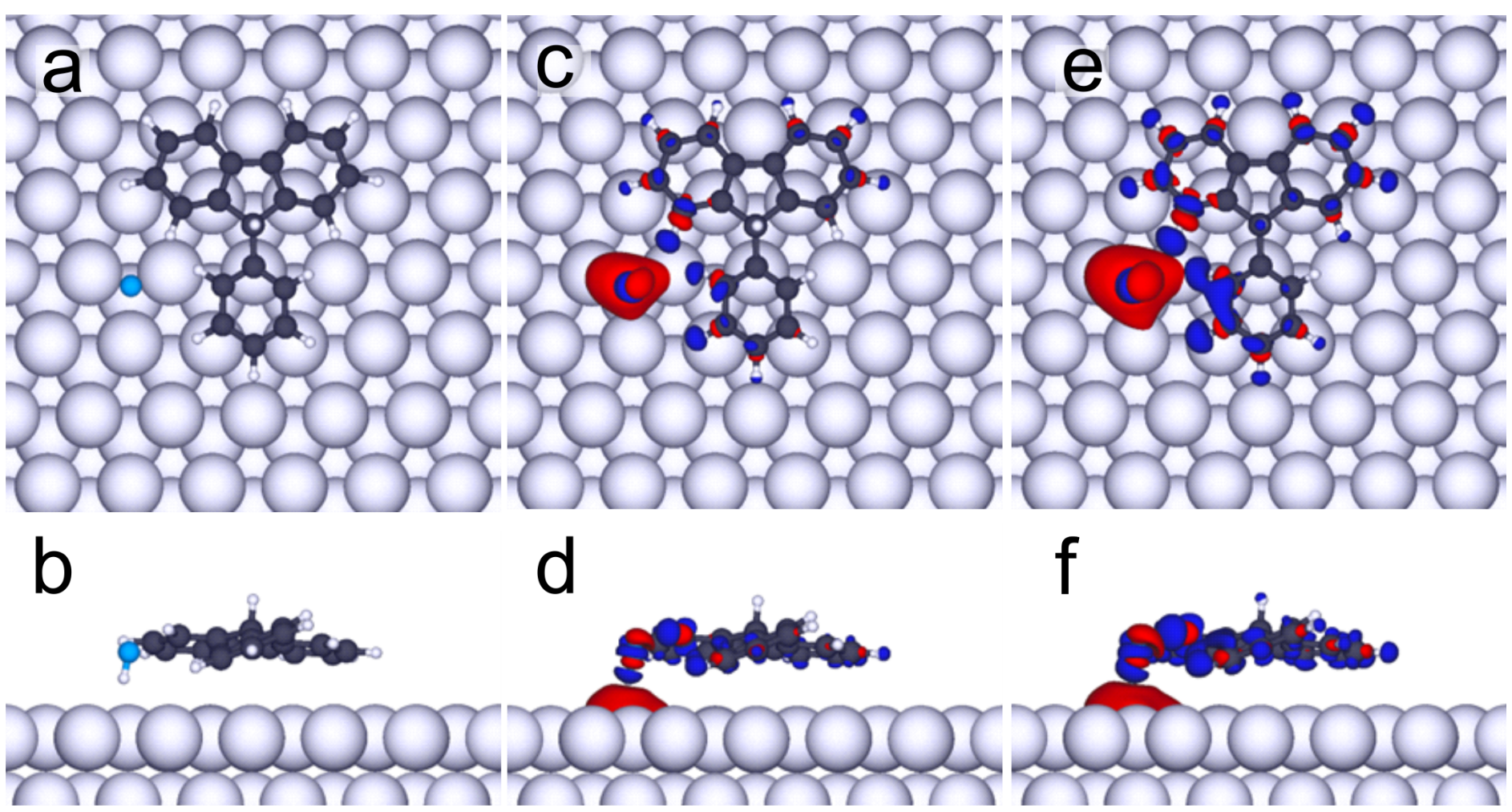

Figure S19: (a,c,e) Top views and (b,d,f) side views of the calculated geometry (a,b) and the CDD isosurfaces (c to f) of product **3b**. (c-f) CDD while treating the HF molecule as a separate component of the charge density of the adsorbate. The isovalues are ± 0.009 e/Å^3 (c,d) and ± 0.006 e/Å^3 (e,f).

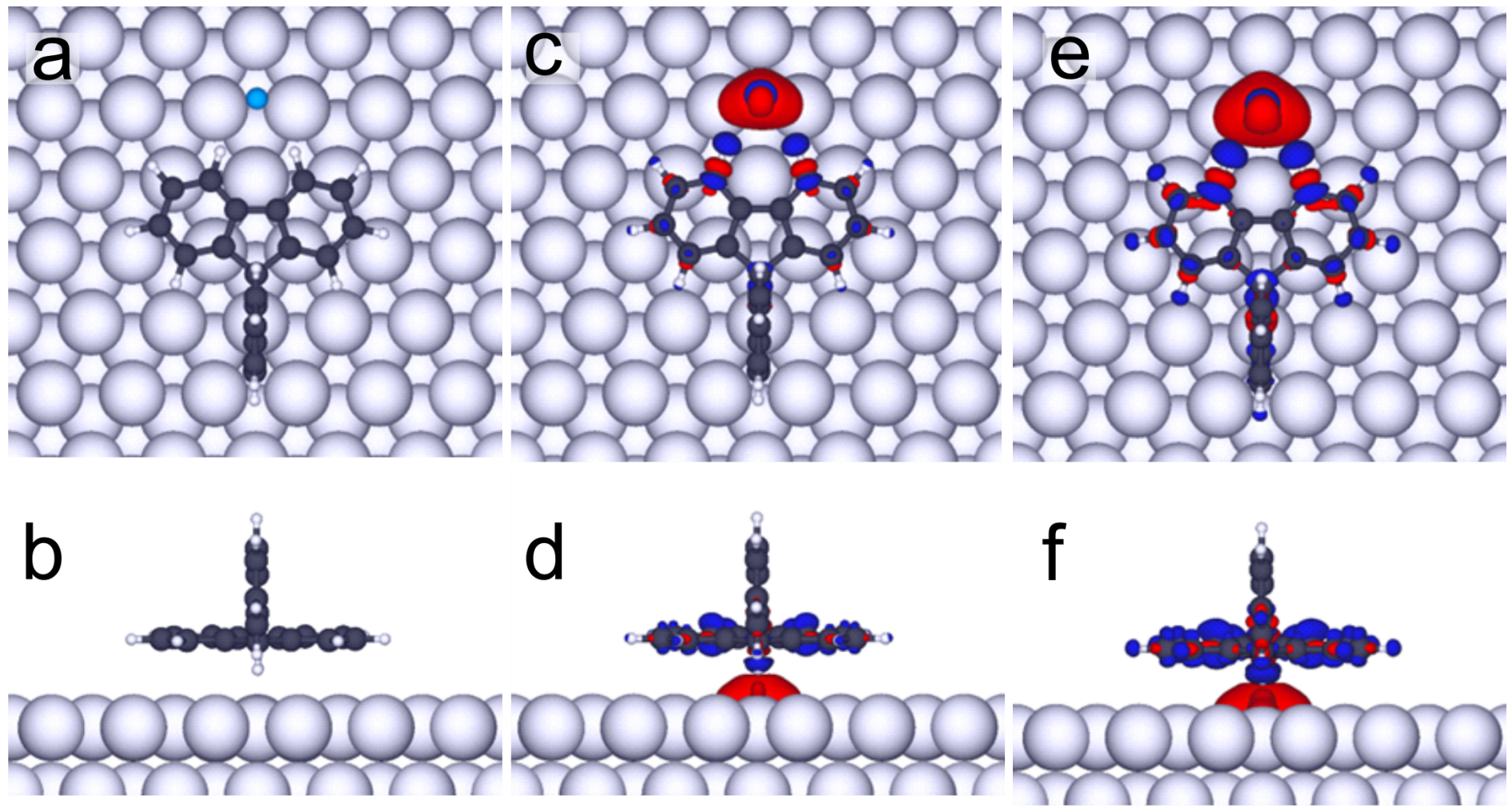

Figure S20: (a,c,e) Top views and (b,d,f) side views of the calculated geometry (a,b) and the CDD isosurfaces (c to f) of product **3c**. (c-f) CDD while treating the HF molecule as a separate component of the charge density of the adsorbate. The isovalues are ± 0.009 e/Å^3 (c,d) and ± 0.006 e/Å^3 (e,f).

Lastly, the CDD isosurfaces of the product structures **3a**, **3b**, and **3c** reveal minor charge modulation between the surface and the molecule (Figs. S18, S19, and S20). However, the phenyl ring, when planar with the Ag(111) surface in product **3b**, transfers more charge on one side than the other with the surface. This is because one side of the phenyl ring lies closer to the surface than the other in order to alleviate steric repulsion within the molecule.

S8.4 Energetics of reactant, intermediate complex, product, and HF

The adsorption energy and binding energy of the reactant **1a** with the fluorine pointing away from Ag(111) were calculated to be −1.28 eV and −1.46 eV, respectively. The adsorption energy and binding energy of the reactant **1b** with the fluorine pointing down on Ag(111) were calculated to be −0.97 eV and −1.11 eV, respectively. When the phenyl ring is rotated in reactant **1c**, the adsorption energy and binding energy lower to −0.79 eV and −0.93 eV. When the fluorine is formed immediately following C-F bond dissociation in intermediate complex **2a**, the adsorption energy is −0.70 eV but the binding energy increases

to a substantial −5.65 eV due in large part to the significant energy associated with fluorine chemisorption.

Table 1: Energetics of reactant **1** and C-F bond dissociation transition state **TS1** on Ag(111). **TS1**$_H$ and **TS1**$_B$ are the transition states for C-F bond dissociation to hollow and bridge site, respectively. E_{rel} is the relative energy, E_{ads} is the adsorption energy, E_b is the binding energy, and iCOHP is the integrated crystal orbital Hamilton population of the C-F bond.

Structure	E_{rel} (eV)	E_{ads} (eV)	E_b (eV)	iCOHP (eV)
**1a**	0	-1.28	-1.46	-8.72
**1b**	0.31	-0.97	-1.11	-7.88
**1c**	0.49	-0.79	-0.93	-6.94
**TS1**$_H$	0.65	-2.78	-5.58	-0.11
**TS1**$_B$	0.73	-2.70	-5.50	-0.41

The chemisorbed intermediate complex **2b** has an adsorption energy of −3.40 eV and a binding energy of −6.19 eV, which clearly shows the strong interaction between the adsorbate and Ag(111). The Ag-C iCOHP for the intermediate complex **2b** is −1.61 eV. Without the fluorine, the binding energy is calculated to be −1.76 eV and proves how influential the fluorine is on stabilizing the intermediate complex. When the Ag-C interaction is broken in intermediate complex **2c**, the adsorption energy reduces to −3.09 eV and the binding energy lowers to −5.88 eV. Other adsorption sites of the intermediate structures and structures without the fluorine atom were also calculated.

Table 2: Energetics of intermediate complex structures **2** on Ag(111). **2b**$_1$ and **2c**$_1$ are the structures with the fluorine atoms moved away, **2b**$_2$ is without the fluorine atom, **2b**$_3$ and **2c**$_2$ are with the central carbons adsorbed to bridge sites, **2b**$_4$ and **2c**$_3$ are with the central carbon adsorbed to H_{fcc} sites, **2b**$_5$ is with the central carbon adsorbed to H_{hcp} site, **2b**$_6$ is rotated by 30°, and **2c**$_4$ is with the central carbon adsorbed to H_{fcc} site without fluorine atom. E_{rel} is the relative energy, E_{ads} is the adsorption energy, and E_b is the binding energy.

Structure	E_{rel} (eV)	E_{ads} (eV)	E_b (eV)
**2b**	0	-3.40	-6.19
**2b**$_1$	0.12	-3.28	-6.08
**2b**$_2$	0	–	-1.76
**2b**$_3$	0.01	-3.39	-6.19
**2b**$_4$	0.13	-3.26	-6.06
**2b**$_5$	0.08	-3.32	-6.12
**2b**$_6$	0.01	-3.39	-6.19
**2c**	0.31	-3.09	-5.88
**2c**$_1$	0.46	-2.93	-5.73
**2c**$_2$	0.32	-3.07	-5.87
**2c**$_3$	0.27	-3.13	-5.93
**2c**$_4$	0.29	–	-1.47

The C-C coupling transition state **TS2** is calculated relative to intermediate complex **2b**. After C-C coupling, the adsorption energy of product **3-H** is −3.20 eV and the binding energy is −6.07 eV, which is slightly stronger than that of intermediate complex **2c** and helps to support why Friedel-Crafts alkylation takes place.

Table 3: Energetics of product **3-H** and **3a** and transition state **TS2** structures on Ag(111). **TS2**$_1$ and **3-H**$_2$ are the structures without the fluorine atom, **3-H**$_1$ is with the fluorine atom far from the reaction site, and **3a**$_1$ is with the hydrogen adsorbed to the Ag(111) surface. E_{rel} is the relative energy, E_{ads} is the adsorption energy, and E_b is the binding energy. E_{rel} of **TS2**, **TS2**$_1$, and **3-H**$_2$ are calculated with respect to intermediate **2b** with and without fluorine atom, as required.

Structure	E_{rel} (eV)	E_{ads} (eV)	E_b (eV)
**TS2**	0.96	-2.44	-5.23
**TS2**$_1$	1.02	-0.73	–
**3-H**	0.83	-3.20	-6.07
**3-H**$_1$	0.76	-3.27	-6.14
**3-H**$_2$	0.45	-1.62	–
**3a**	0	-1.47	-1.61
**3a**$_1$	0.20	-1.26	-1.40

The planar product **3b** has an adsorption energy of −1.39 eV. The product **3c** with the phenyl rotated perpendicular to the surface has an adsorption energy of −1.31 eV. With the HF molecule still adsorbed on the surface, the adsorption energy and binding energy of product **3b** are −1.63 eV and −1.78 eV, respectively. With the HF molecule still adsorbed on the surface, the adsorption energy and binding energy of product **3c** are −1.60 eV and −1.67 eV, respectively.

Table 4: Energetics of product **3b** and **3c** on Ag(111). $\mathbf{3b}_1$ and $\mathbf{3c}_1$ are the structures without HF molecules. E_{rel} is the relative energy, E_{ads} is the adsorption energy, and E_b is the binding energy.

Structure	E_{rel} (eV)	E_{ads} (eV)	E_b (eV)
**3b**	0.16	-1.63	-1.78
$\mathbf{3b}_1$	0.19	-1.39	–
**3c**	0	-1.60	-1.67
$\mathbf{3c}_1$	0	-1.31	–

Lastly, and because it is a major byproduct of the reaction, we look to HF adsorption and its energetics to explain why we do not seem to see HF in the XPS or in the STM images. Direct adsorption of HF to the Ag(111) surface prefers pointing the hydrogen toward the Ag atoms, but the adsorption energy is only totaled to be −0.22 eV. The Ag-HF iCOHP is -0.96 eV. This is a weak adsorption and supports why it desorbs readily at the above-room temperatures used in the experiments. The H-F bond is calculated to be strong at −8.10 eV from its iCOHP. Dissociated HF is calculated to have a binding energy of −7.37 eV, while the binding energy for the bonded HF molecule is calculated to be −7.77 eV, which supports the observation that HF will form from chemisorbed hydrogen and fluorine meeting at the surface.

Table 5: Energetics of HF adsorption and dissociation on Ag(111). $HF_{(1)}$ is the structure with the hydrogen downward-facing, $HF_{(2)}$ is with the hydrogen tilted downward, $HF_{(3)}$ is with the hydrogen upward-facing, and $HF_{(4)}$ is the dissociated HF with a H—F Distance of 4.5 Å. E_{rel} is the relative energy, E_{ads} is the adsorption energy, E_b is the binding energy, q_F is the charge of the fluorine atom, and q_H is the charge of the hydrogen atom.

Structure	E_{rel} (eV)	E_{ads} (eV)	E_b (eV)	q_F (e)	q_H (e)
$HF_{(1)}$	0	0.22	-7.77	-0.76	+0.68
$HF_{(2)}$	0.02	-0.20	–7.75	–	–
$HF_{(3)}$	0.08	-0.14	-7.69	–	–
$HF_{(4)}$	0.40	0.18	-7.37	-0.69	-0.20

S8.5 C-H deprotonation into the final product

Deprotonation to the Ag(111) surface from the C-C coupled carbocation is computed to be only 0.20 eV less energetically preferred relative to deprotonating directly to the chemisorbed fluorine atom. This suggests that C-C coupling is the most difficult part of the reaction since the C-H deprotonation to form the final product can trigger regardless of whether the fluorine is nearby the reaction site or not. The importance of being able to deprotonate the C-H into Ag-H to make the product is reinforced by the finding that the fluorine atom prefers adsorption away from the reactive C-H site by 0.07 eV.

Table 6: Energetics of deprotonation pathways and C-C coupled carbocation structures **3-H** on Ag(111). **3-H**$_1$ is the structure with the fluorine atom far away from the reaction site, **3-H**$_{depr(1)}$ is with the ring deprotonated prior to C-C coupling, **3-H**$_{depr(2)}$ is with the central carbon deprotonated prior to C-C coupling, **3-H**$_{depr(3)}$ is with the central carbon deprotonated after C-C coupling, and **3a** is with the deprotonation reforming the aromatic ring directly after C-C coupling. E_{rel} is the relative energy, and E_{ads} is the adsorption energy.

Structure	E_{rel} (eV)	E_{ads} (eV)
**3-H**	0.07	-3.20
**3-H**$_1$	0	-3.27
**3-H**$_{depr(1)}$	0.96	-3.31
**3-H**$_{depr(2)}$	1.20	-2.73
**3-H**$_{depr(3)}$	0.48	-1.93
**3a**	-0.76	-1.47

S8.6 Charge distributions in the reactant, intermediate complex, and product

Adsorption of reactant **1a** on the Ag(111) surface results in a slight charge transfer from the surface to the molecule, yielding a net molecular charge of -0.02 e. In contrast, reactant **1b** exhibits a more pronounced charge transfer, with the molecule acquiring a net charge of -0.07 e. This difference arises in part from the fluorine atom, which becomes more negatively charged (-0.64 e) in **1b** compared to **1a** (-0.61 e). Correspondingly, the carbon framework of the reactant carries a net positive charge of +0.59 e (**1a**) and +0.56 e (**1b**), indicating that the organic moiety is positively charged even prior to C-F bond dissociation. Upon C-F bond dissociation, the resulting intermediate complex **2a** features a chemisorbed fluorine with a charge of -0.71 e and a molecular fragment with a net charge of +0.21 e. These values are consistent with those obtained for the nudged elastic band transition state **TS1**, supporting the conclusion that the molecular fragment adopts a partially positive charge following bond dissociation.

Table 7: Charge distribution for reactants **1**, C-F bond dissociation transition states **TS1**, and the intermediate complex **2a**. **TS1**$_H$ and **TS1**$_B$ are the transition states for C-F bond dissociation to hollow and bridge sites, respectively. q_F is the charge of the fluorine atom, q_{Mol} the charge of the molecule, q_{Mol+F} is the charge of the molecule and the fluorine atom, $q_{F(gp)}$ is the charge of the fluorine in the gas-phase, and $q_{Mol(gp)}$ is the charge of the molecule in the gas-phase.

Structure	q_F (e)	q_{Mol} (e)	q_{Mol+F} (e)	$q_{F(gp)}$ (e)	$q_{Mol(gp)}$ (e)
**1a**	-0.61	+0.59	-0.02	-0.62	+0.62
**1b**	-0.64	+0.56	-0.07	-0.60	+0.60
**1c**	-0.63	+0.56	-0.07	-0.58	+0.58
**TS1**$_H$	-0.71	+0.20	-0.51	-0.67	+0.67
**TS1**$_B$	-0.69	+0.34	-0.35	-0.62	+0.62
**2a**	-0.71	+0.21	-0.50	-0.67	+0.67

In intermediate complex **2b** where the fluorine resides beneath the biphenyl ring, the fluorine retains a charge of -0.71 e, while the molecular fragment becomes slightly negatively charged (-0.16 e). Charge density difference (CDD) analysis reveals electron accumulation

between the Ag(111) surface top site and the central carbon atom, rationalizing this charge redistribution. Removal of the fluorine from the surface leads to an intermediate with an increased negative charge (-0.23 e), indicating that the fluorine withdraws electron density from the molecule.

In this negatively charged state, the molecule is not predisposed toward Friedel-Crafts alkylation. However, upon weakening of the Ag-C interaction in intermediate complex **2c**, the molecular fragment becomes positively charged (+0.07 e), while the fluorine remains unchanged (-0.71 e). At alternative adsorption sites, where the central carbon is lifted further from the surface, the positive charge on the molecule increases to +0.09 e (bridge site) and +0.10 e (hollow site). These observations indicate that weakening the Ag-C interaction is a prerequisite for generating the positively charged character of the central carbon atom necessary for C-C bond formation.

Table 8: Charge distribution for intermediate complex structures **2** on Ag(111). $\mathbf{2b}_1$ and $\mathbf{2c}_1$ are the structures with the fluorine atoms moved away, $\mathbf{2b}_2$ is without the fluorine atom, $\mathbf{2b}_3$ and $\mathbf{2c}_2$ are with the central carbons adsorbed to bridge sites, $\mathbf{2b}_4$ and $\mathbf{2c}_3$ are with the central carbon adsorbed to H_{fcc} sites, $\mathbf{2b}_5$ is with the central carbon adsorbed to H_{hcp} site, $\mathbf{2b}_6$ is rotated by 30°, and $\mathbf{2c}_4$ is with the central carbon adsorbed to H_{fcc} site without fluorine atom. q_F is the charge of the fluorine atom, q_{Mol} the charge of the molecule, q_{Mol+F} is the charge of the molecule and the fluorine atom, $q_{F(gp)}$ is the charge of the fluorine in the gas-phase, and $q_{Mol(gp)}$ is the charge of the molecule in the gas-phase.

Structure	q_F (e)	q_{Mol} (e)	q_{Mol+F} (e)	$q_{F(gp)}$ (e)	$q_{Mol(gp)}$ (e)
**2b**	-0.71	-0.16	-0.86	-0.62	0.62
$\mathbf{2b}_1$	-0.70	-0.17	-0.87	-0.51	0.51
$\mathbf{2b}_2$	–	-0.23	-0.23	–	–
$\mathbf{2b}_3$	-0.71	-0.10	-0.81	-0.62	0.62
$\mathbf{2b}_4$	-0.72	-0.06	-0.78	-0.66	0.66
$\mathbf{2b}_5$	-0.72	-0.06	-0.78	-0.66	0.66
$\mathbf{2b}_6$	-0.72	-0.13	-0.85	-0.64	0.64
**2c**	-0.71	0.07	-0.64	-0.60	0.60
$\mathbf{2c}_1$	-0.70	0.02	-0.68	-0.52	0.52
$\mathbf{2c}_2$	-0.72	0.09	-0.63	-0.63	0.63
$\mathbf{2c}_3$	-0.71	0.10	-0.62	-0.64	0.64
$\mathbf{2c}_4$	–	-0.01	-0.01	–	–

This conclusion is further supported by the charge states of the C-C coupled species: the

C-C coupled product **3-H** exhibits a net charge of +0.18 e, and the corresponding transition state **TS2** reaches +0.24 e, underscoring the requirement for a positive charge. Subsequent deprotonation neutralizes the product in **3a** and forms HF, which carries a charge of -0.08 e.

Table 9: Charge distribution for product **3-H** and **3a** and transition state **TS2** structures. **TS2**$_1$ and **3-H**$_2$ are the structures without the fluorine atom, **3-H**$_1$ is with the fluorine atom far from the reaction site, and **3a**$_1$ is with the hydrogen adsorbed to the Ag(111) surface. q_{HF} is the charge of HF, q_{Mol} the charge of the molecule, q_{HF+Mol} is the charge of HF and the molecule, $q_{HF(gp)}$ is the charge of HF in the gas-phase, and $q_{Mol(gp)}$ is the charge of the molecule in the gas-phase.

Structure	q_{HF} (e)	q_{Mol} (e)	q_{HF+Mol} (e)	$q_{HF(gp)}$ (e)	$q_{Mol(gp)}$ (e)
**TS2**	-0.72	0.24	-0.48	-0.64	0.64
**TS2**$_1$	N/A	0.18	0.18	N/A	N/A
**3-H**	-0.71	0.18	-0.52	-0.64	0.64
**3-H**$_1$	-0.72	0.17	-0.56	-0.63	0.63
**3-H**$_2$	N/A	0.09	0.09	N/A	N/A
**3a**	-0.08	0.00	-0.08	-0.05	0.05
**3a**$_1$	-0.92	0.04	-0.88	-0.52	0.52

In the presence of HF, the products exhibit small residual charges of +0.04 e (**3b**) and +0.02 e (**3c**). Upon HF desorption, these charges further diminish to +0.01 e and -0.02 e, respectively, consistent with the enhanced stability of the final products relative to the reactive intermediates.

Table 10: Charge distribution for final product **3b** and **3c**. **3b**$_1$ and **3c**$_1$ are the structures without HF. q_{HF} is the charge of HF, q_{Mol} the charge of the molecule, q_{HF+Mol} is the charge of HF and the molecule, $q_{HF(gp)}$ is the charge of HF in the gas-phase, and $q_{Mol(gp)}$ is the charge of the molecule in the gas-phase.

Structure	q_{HF} (e)	q_{Mol} (e)	q_{HF+Mol} (e)	$q_{HF(gp)}$ (e)	$q_{Mol(gp)}$ (e)
**3b**	-0.10	0.04	-0.06	0	0
**3b**$_1$	N/A	0.01	0.01	N/A	N/A
**3c**	-0.10	0.02	-0.08	0	0
**3c**$_1$	N/A	-0.02	-0.02	N/A	N/A

S8.7 Hexamers of the intermediate complex

The intermediate clusters found experimentally are predominantly hexamers. The reason for the clustering of the intermediate complexes is to force the fluorine atoms to interact with

more than just one molecule through ion-dipole forces.

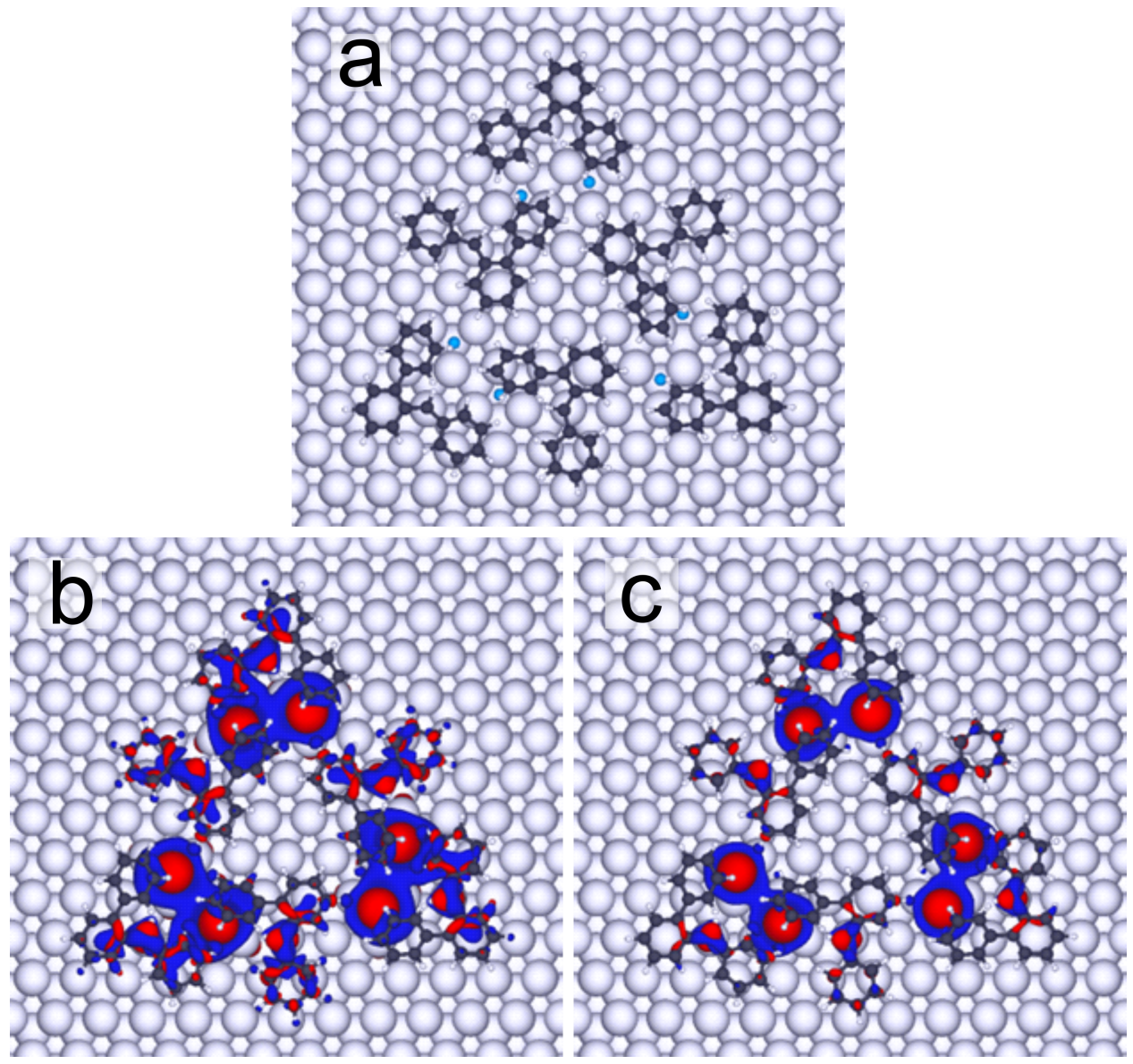

Figure S21: (a) Top view of the calculated geometry and (b,c) CDD isosurfaces of the intermediate complex **2b** hexamers. (b,c) CDD while treating the fluorine as a separate component of the charge density of the adsorbate. The isovalues are (b) ±0.006 e/$Å^3$ and (c) ±0.009 e/$Å^3$.

These interactions are notable in the CDD isosurfaces through more charge loss on C-H groups of the molecules. Furthermore, the hexamer intermediate complex clusters place nearby fluorine atoms at position 4 hollow sites relative to one another, which was observed to be the preferred positioning for chlorine on Ag(111) previously[17] and was found to be what is preferred for the fluorine dimer adsorbed to Ag(111).

Given the results from the FPMD showing that the intermediate monomer is difficult to make react at 375 K even after 15 ps, we speculate that clustering may assist the intermediate to react by breaking apart the Ag-C interaction to form the reactive positively charged intermediate complex.

To assess the hexamer intermediate's reactivity, we explored C-C coupling for one interior intermediate complex and one exterior intermediate complex in the hexamer cluster. Performing C-C coupling in the exterior intermediate complex is calculated to be the most efficient as the C-C coupled cation is only 0.39 eV less stable than the chemisorbed interme-

diate complex, which is in contrast to the difference of 0.44 eV for the monomer. However, C-C coupling in the interior intermediate complex is severely limited as the difference is now 0.74 eV when compared to the intermediate complex. Therefore, the hexamer cluster enhances reactivity in the exterior intermediates but makes it so the interior intermediate complexes of the cluster have heightened stability.

Table 11: Energetics of **2b** hexamer structures $\mathbf{2b}_{Hexa}$ and C-C bond formations on Ag(111). $\mathbf{2b}_{Hexa(1)}$ is the hexamer with an outer molecular C-C bond formation and $\mathbf{2b}_{Hexa(2)}$ with an inner. $\mathbf{2b}_{Hexa(3)}$ is the hexamer with an outer product and $\mathbf{2b}_{Hexa(4)}$ with an inner. E_{rel} is the relative energy, E_{ads} is the adsorption energy, E_b is the binding energy, E_{ads}/Mol is the adsorption energy per molecule, and E_b/Mol is the binding energy per molecule.

Structure	E_{rel} (eV)	E_{ads} (eV)	E_b (eV)	E_{ads}/Mol (eV/M)	E_b/Mol (eV/M)
$\mathbf{2b}_{Hexa}$	0	-20.92	-37.72	-3.49	-6.29
$\mathbf{2b}_{Hexa(1)}$	0.39	-20.78	-37.64	-3.46	-6.27
$\mathbf{2b}_{Hexa(2)}$	0.74	-20.43	-37.29	-3.40	-6.22
$\mathbf{2b}_{Hexa(3)}$	-1.09	-19.70	-33.84	-3.28	-5.64
$\mathbf{2b}_{Hexa(4)}$	-0.95	-19.55	-33.69	-3.26	-5.62

The charge/molecule of the intermediate complex in the carbon-based molecule is -0.18 e/molecule for the dimer and -0.17 e/molecule for the hexamer. Relative to the intermediate complex monomer (-0.16 e), this shows that clustering further enhances placing a negative charge on the molecule and deactivating the Friedel-Crafts reaction.

S8.8 Energetics for the reaction on the surface vs gas phase

Full C-F bond dissociation requires 7.64 eV at most or as little as 2.01 eV in the gas phase. Bringing in the fluorine ion to be nearby the carbocation is stabilizing and releases 4.31 eV of energy. The Ag(111) surface has a major impact on the reaction as it makes it so the C-F bond dissociation step requires less energy (0.65 eV) when compared to the C-C coupling step (0.96 eV). In the gas-phase, C-C coupling requires as little as 0.41 eV, which is the least amount of energy when considering all major reaction steps in the gas-phase or on the surface. Therefore, the rate-determining step is C-C coupling on the surface, reinforcing the ability for the surface to isolate the intermediate complex, but C-F bond dissociation

is rate-determining in the gas-phase, suggesting that the intermediate complex may not be isolated in the gas phase.

In the gas phase, a transition state could not be easily identified for C-F bond dissociation or for the entire reaction to take place in one single step. The transition state reported upon in the gas phase for C-F bond dissociation was obtained from restricting the movement of the fluorine atom with respect to the sp^3 hybridized carbon. Gas-phase transition states were identified for protonating the product back into the intermediate complex and for C-C coupling.

Attempts were made to optimize the C-C coupling transition state from the $C_{19}H_{15}$ intermediate by treating it as either a carbocation or a neutral/radical species. By treating the $C_{19}H_{15}$ intermediate as a carbocation, the transition state optimization pushed the intermediate into successfully identifying the true transition state to C-C coupling with an activation energy of 0.41 eV and the protonated C-C coupled species being only 0.03 eV less stable than the uncoupled carbocation intermediate. When treating the $C_{19}H_{15}$ intermediate as a neutral/radical species and optimizing for the transition state, the phenyl ring carbon was observed to repel the reactive carbon site. This occurs due to the phenyl ring being incapable of acting as a nucleophile with the reactive carbon because it takes on a partial negative charge from the radical electron largely being present there. This finding proves the importance of the intermediate carbon-based molecule localizing a positive charge on itself in order to form an intramolecular cyclized C-C bond. On the surface, the intermediate $C_{19}H_{15}$ takes on a partial negative charge but regains its positive charge when breaking from the Ag(111) surface. This supports the capability of isolating $C_{19}H_{15}$ and chemisorbed fluorine prior to completing the cyclization reaction by forcing a negative charge onto the molecule. In this work, this is performed through charge transfer from the underlying Ag(111) surface to the $C_{19}H_{15}$. Therefore, breaking the carbon to silver interaction is crucial to destabilize the molecule into the carbocation for the Friedel-Crafts alkylation reaction to succeed, but also shows that the surface can greatly stabilize a reactive carbon-based molecule through

significant charge transfer and bonding.

The transition state to C-C coupling for the carbocation intermediate shows a singular negative imaginary frequency at -296.32 cm^{-1} / -282.67 cm^{-1} with an intensity of 419.62 / 441.57 in the gas phase / surface. The negative imaginary frequency has solely C-C bond formation character to the vibration.

Attempts were made and met with failure to locate the transition state to C-H deprotonation in the gas-phase and on Ag(111) to directly form a HF molecule or transfer H^+ directly to the Ag(111) surface. From this finding, we conclude that deprotonating the $C_{19}H_{15}^+$ C-C coupled species with F^- or Ag(111) into the product $C_{19}H_{14}$ and HF does not have a substantive barrier in the gas phase or on the Ag(111) surface. This is due to the molecule greatly favoring reformation of its aromaticity and the hydrogen atom being capable of tunneling with ease.

Surface-adsorbed C-F bond dissociation transition states show a negative imaginary frequency at -210.94 cm^{-1} / -153.56 cm^{-1} with an intensity of 78.43 / 33.19 for the F atom moving to a bridge site / hollow site. In the gas phase, two C-F bond dissociation transition states were found which show a single negative imaginary frequency each at either -198.16 cm^{-1} or -218.67 cm^{-1} depending on the fluorine location relative to the sp^3 hybridized carbon.

Table 12: Comparison of VASP-calculated relative energies for key structures on the Ag(111) surface and in the gas phase. $E_{rel(surf)}$ is the surface relative energy and $E_{rel(gp)}$ is the gas-phase relative energy. Energies are referenced to the most stable configuration in each environment, being product **3c**.

Structure	$E_{rel(surf)}$ (eV)	$E_{rel(gp)}$ (eV)
**1a**	0.69	0.56
**1b**	1.00	0.60
**1c**	1.18	0.86
**TS1**	1.33	2.71
**2a**	1.27	2.94
**2b**	0.72	3.43
**2c**	1.03	3.28
**TS2**	1.68	3.58
**3-H**	1.16	3.32
**3a**	0.33	0.09
**3b**	0.16	0.39
**3c**	0.00	0.00

S8.9 Energetics for hydrogenated, chlorinated, brominated, and iodinated reactants

Table 13: Energetics of the structures for the reaction of the fluorinated reactant on Ag(111). E_{rel} is the relative energy to reactant **1a**, E_{ads} is the adsorption energy, and E_b is the binding energy.

Structure (H)	E_{rel} (eV)	E_{ads} (eV)	E_b (eV)
**1a**	0	-1.28	-6.45
**1b**	0.31	-0.97	-6.14
**1c**	0.49	-0.79	-5.96
**TS1**	0.65	-0.64	-5.80
**2a**	0.58	-0.70	-5.87
**2b**	0.03	-1.25	-6.42
**2c**	0.34	-0.94	-6.11
**TS2**	0.99	-0.29	-5.46
**3-H**	0.47	-0.81	-5.98
**3a**	-0.35	-1.64	-6.80
**3b**	-0.52	-1.81	-6.97
**3c**	-0.69	-1.97	-7.13

Table 14: Energetics of the structures for the reaction of the hydrogenated reactant on Ag(111). E_{rel} is the relative energy, E_{ads} is the adsorption energy, and E_b is the binding energy.

Structure (H)	E_{rel} (eV)	E_{ads} (eV)	E_b (eV)
**1a**	0	-1.36	-6.23
**1b**	0.23	-1.12	-6.00
**1c**	0.35	-1.01	-5.89
**TS1**	–	–	–
**2a**	1.58	0.22	-4.66
**2b**	1.20	-0.16	-5.04
**2c**	1.52	0.16	-4.72
**TS2**	–	–	–
**3-H**	1.64	0.28	-4.60
**3a**	0.71	-0.65	-5.53
**3b**	0.54	-0.81	-5.69
**3c**	0.38	-0.98	-5.86

Table 15: Energetics of the structures for the reaction of the chlorinated reactant on Ag(111). E_{rel} is the relative energy, E_{ads} is the adsorption energy, and E_b is the binding energy.

Structure (Cl)	E_{rel} (eV)	E_{ads} (eV)	E_b (eV)
**1a**	0	-1.36	-4.74
**1b**	0.50	-0.86	-4.24
**1c**	–	–	–
**TS1**	–	–	–
**2a**	0.15	-1.21	-4.59
**2b**	-0.67	-2.03	-5.41
**2c**	-0.35	-1.71	-5.09
**TS2**	–	–	–
**3-H**	-0.25	-1.61	-4.99
**3a**	-0.38	-1.75	-5.12
**3b**	-0.40	-1.76	-5.14
**3c**	-0.58	-1.94	-5.32

Table 16: Energetics of the structures for the reaction of the brominated reactant on Ag(111). E_{rel} is the relative energy, E_{ads} is the adsorption energy, and E_b is the binding energy.

Structure (Br)	E_{rel} (eV)	E_{ads} (eV)	E_b (eV)
**1a**	0	-1.42	-4.18
**1b**	-0.16	-1.58	-4.34
**1c**	–	–	– -
**TS1**	–	–	–
**2a**	-0.39	-1.81	-4.57
**2b**	-0.88	-2.31	-5.07
**2c**	-0.54	-1.97	-4.73
**TS2**	–	–	–
**3-H**	-0.48	-1.90	-4.66
**3a**	-0.27	-1.69	-4.45
**3b**	-0.29	-1.71	-4.47
**3c**	-0.48	-1.90	-4.66

Table 17: Energetics of the structures for the reaction of the iodinated reactant on Ag(111). E_{rel} is the relative energy, E_{ads} is the adsorption energy, and E_b is the binding energy.

Structure (I)	E_{rel} (eV)	E_{ads} (eV)	E_b (eV)
**1a**	0	-1.48	-3.69
**1b**	-0.46	-1.93	-4.15
**1c**	–	–	–
**TS1**	–	–	–
**2a**	-0.70	-2.18	-4.39
**2b**	-1.07	-2.54	-4.76
**2c**	-0.70	-2.17	-4.39
**TS2**	–	–	–
**3-H**	-0.66	-2.14	-4.35
**3a**	-0.21	-1.68	-3.90
**3b**	-0.23	-1.71	-3.92
**3c**	-0.42	-1.89	-4.11

In order to better understand the effects from using fluorine as the halogen in the on-surface Friedel-Crafts alkylation reaction, computations were performed on the structures relevant to the Friedel-Crafts alkylation while replacing fluorine with chlorine, bromine, iodine, and hydrogen. When hydrogen was used instead of fluorine, the energetics of the reaction reveal that, of all the structures, the reactant would remain as it is most preferable. In fact, when attempting to dissociate hydrogen instead of fluorine to turn 1a into 2b but with

the hydrogen adsorbed to Ag(111) and the fluorine staying bonded to the central carbon, the reactant structure was energetically preferred by 1.67 eV relative to the intermediate complex. Thus, hydrogen is unable to replace fluorine or dissociate to facilitate the initial stage of the reaction.

When using chlorine, bromine, and iodine, we found that the Cl, Br, and I prefer to remain dissociated on the Ag(111) surface instead of forming HCl, HBr, or HI and then desorbing thereafter as HF is capable of doing. With HF, the bonding energy is strong enough between the two atoms that its formation assists in pulling the chemisorbed fluorine atom off the surface after it acts like a base with the activated C-H entity. Our computations show that the Ag(111) surface acts as a stronger base than the chemisorbed chlorine, bromine, and iodine do, which contrasts with what is seen with fluorine. Furthermore, removing Cl, Br, and I can be an issue in on-surface synthesis as it necessitates higher temperatures. This natural preference to cleaning the surface of chemisorbed fluorine by forming HF and leaving behind C-C coupled product may be useful to on-surface synthesis with halogenated precursors. Therefore, C-C coupled product formation is preferred and attainable when using chlorine, bromine, and iodine, but the halogens will remain chemisorbed to Ag(111).

Prior to C-C coupled product formation, the intermediate complex is shown to grow increasingly more stable with moving down the halogen group of the periodic table. The relative energies of 1a versus 2b for fluorine, chlorine, bromine, and iodine is 0.03 eV, -0.67 eV, -0.88 eV, and -1.07 eV, respectively. Therefore, the intermediate complex is capable of being stabilized by other negatively charged adsorbed species.

An important component to the reaction energetics is the carbon to halogen bond strength and its dissociation to initiate the reaction. Our computations reveal that the C-F bond is strongest in 1a and weakens moving down the halogen group. This trend helps to explain why the C-Br and C-I bonds could not stay intact when building 1b or 1c structures computationally. When comparing precursor structures, C-Cl bonds reveal a less stable 1b structure relative to implementing C-F bonds and the inability to relax into a 1c structure

with chlorine. Dissociating the C-Cl, C-Br, and C-I bonds is more energetically feasible but the C-F bonding strength allows for a more involved reaction and structural analysis of the reactant. Therefore, by studying the most difficult to dissociate C-F bond on the Ag(111) surface and capturing the reactant, intermediate, and product structures, the other halogenated precursor options should make for effective options to on-surface Friedel-Crafts alkylations.

S8.10 Flipping barriers of reactant and product

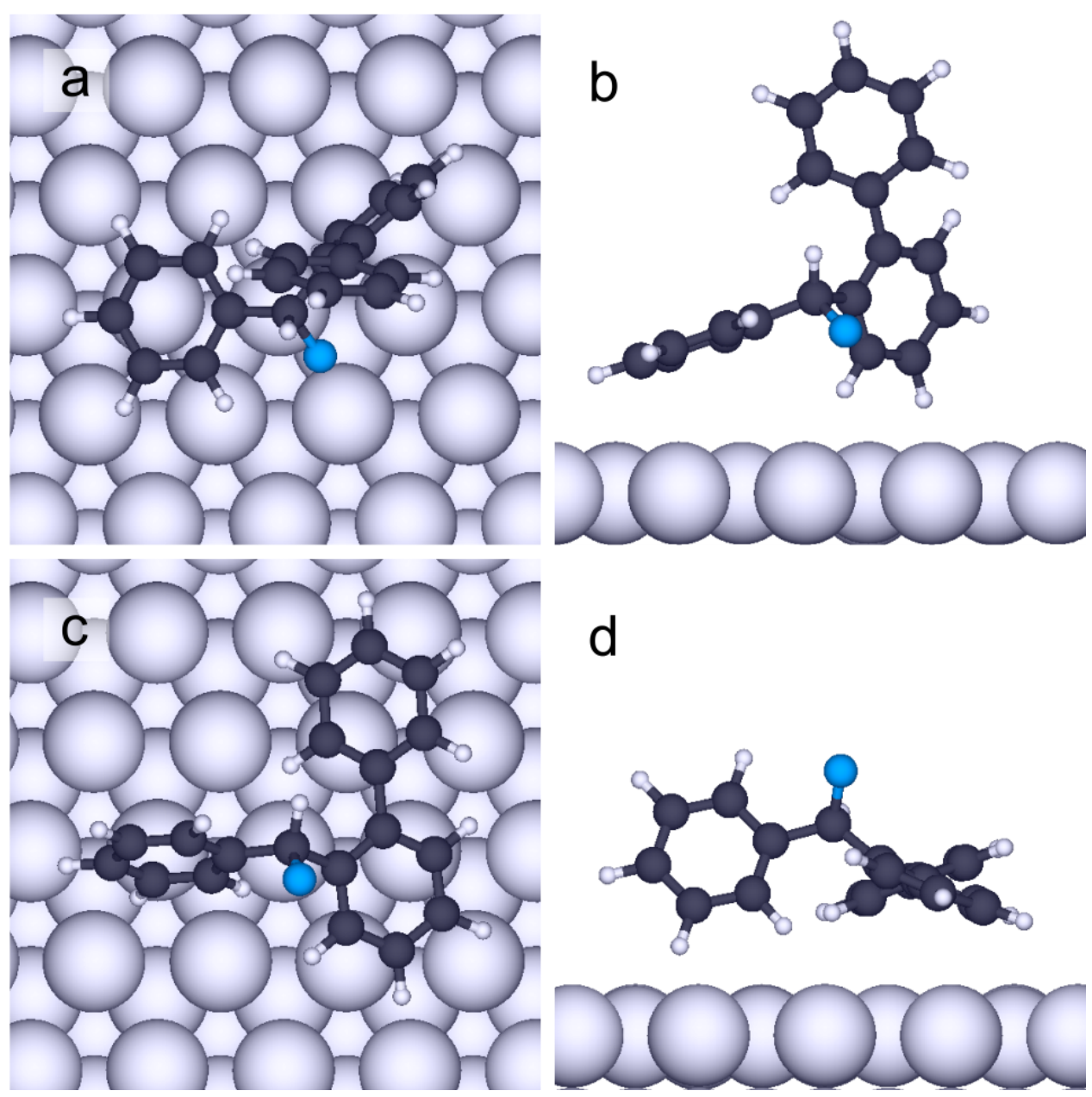

Figure S22: Flipping transition-state for reactant **1a** to **1b**: (a) Top view and (b) side view of the transition-state via flipping the biphenyl. (c) Top view and (d) side view of the transition-state for the rotated single phenyl ring.

In the main text we explain the flipping of reactant **1a** to **1b**. Here we provide the corresponding transition-state structures. The transition proceeding via flipping of the biphenyl unit is shown in Fig. S22a,b. An alternative pathway first rotates the single phenyl ring before rotating the biphenyl. The corresponding structure is shown in Fig. S22c,d.

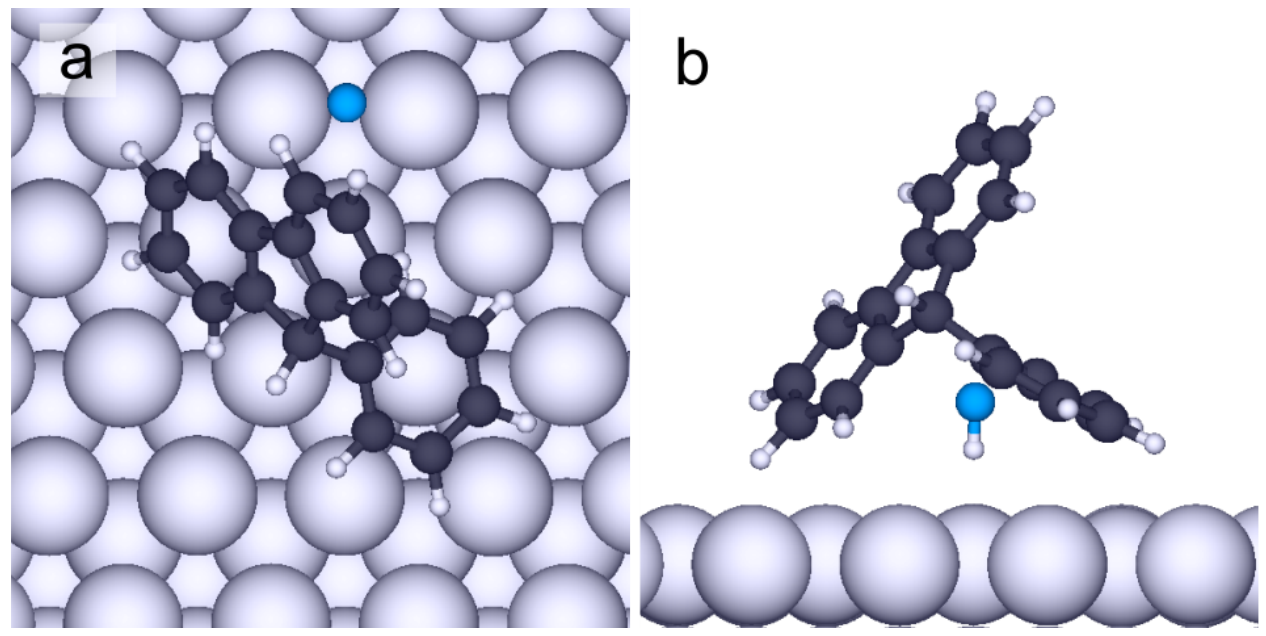

Figure S23: Flipping transition-state for product **3b** to **3c**: (a) Top view and (b) side view of the transition-state.

We further explain the predominance of the planar product **3b** compared to **3c** with a kinetic limitation in the main text. Here we provide the transition-state structure (Fig. S23a,b).

References

(1) Z. Deng, J.-H. Lin, J.-C. Xiao, "Nucleophilic Arylation with Tetraarylphosphonium Salts," Nature Communications 7 (2016): 10337, https://doi.org/10.1038/ncomms10337.

(2) K. Morgenstern, N. Lorente, K.-H. Rieder, "Controlled Manipulation of Single Atoms and Small Molecules Using the Scanning Tunnelling Microscope," Physica Status Solidi B 250 (2013): 1671–1751, https://doi.org/10.1002/pssb.201248392.

(3) I. Horcas, R. Fernández, J. M. Gómez-Rodríguez, J. Colchero, J. Gómez-Herrero, A. M. Baro, "WSXM: A Software for Scanning Probe Microscopy and a Tool for Nanotechnology," Review of Scientific Instruments 78 (2007): 013705, https://doi.org/10.1063/1.2432410.

(4) N. Fairley, V. Fernandez, M. Richard-Plouet, et al., "Systematic and Collaborative Approach to Problem Solving Using X-Ray Photoelectron Spectroscopy," Applied Surface Science Advances 5 (2021): 100112, https://doi.org/10.1016/j.apsadv.2021.100112.

(5) A. G. Shard, ”Practical Guides for X-Ray Photoelectron Spectroscopy: Quantitative XPS,” Journal of Vacuum Science & Technology A 38 (2020): 041201, https://doi.org/10.1116/1.5141395.

(6) G. Kresse, J. Furthmüller, ”Efficient iterative schemes for *ab initio* total-energy calculations using a plane-wave basis set,” Physical Review B 54 (1996): 11169–11186, https://doi.org/10.1103/PhysRevB.54.11169.

(7) J. Klimeš, D. R. Bowler, A. Michaelides, ”Chemical Accuracy for the van der Waals Density Functional,” Journal of Physics: Condensed Matter 22 (2010): 022201, https://doi.org/10.1088/0953-8984/22/2/022201.

(8) J. Klimeš, D. R. Bowler, A. Michaelides, ”Van der Waals density functionals applied to solids,” Physical Review B 83 (2011): 195131, https://doi.org/10.1103/PhysRevB.83.195131.

(9) P. E. Blöchl, ”Projector augmented-wave method,” Physical Review B 50 (1994): 17953–17979, https://doi.org/10.1103/PhysRevB.50.17953.

(10) G. Kresse, D. Joubert, ”From ultrasoft pseudopotentials to the projector augmented-wave method,” Physical Review B 59 (1999): 1758–1775, https://doi.org/10.1103/PhysRevB.59.1758.

(11) H. J. Monkhorst, J. D. Pack, ”Special points for Brillouin-zone integrations,” Physical Review B 13 (1976): 5188–5192, https://doi.org/10.1103/PhysRevB.13.5188.

(12) A. W. Dweydari, C. H. B. Mee, ”Oxygen Adsorption on the (111) Face of Silver,” Physica Status Solidi (a) 17 (1973): 247–250, https://doi.org/10.1002/pssa.2210170128.

(13) S. Maintz, V. L. Deringer, A. L. Tchougréeff, R. Dronskowski, ”LOBSTER: A tool to extract chemical bonding from plane-wave based DFT,” Journal of Computational Chemistry 37 (2016): 1030–1035, https://doi.org/10.1002/jcc.24300.

(14) M. J. Frisch, G. W. Trucks, H. B. Schlegel, et al., *Gaussian 16, Revision C.01*; Gaussian, Inc.: Wallingford, CT, **2016**.

(15) J. P. Perdew, M. Ernzerhof, K. Burke, "Rationale for Mixing Exact Exchange with Density Functional Approximations," The Journal of Chemical Physics 105 (1996): 9982–9985, https://doi.org/10.1063/1.472933.

(16) S. Grimme, J. Antony, S. Ehrlich, H. Krieg, "A Consistent and Accurate *ab Initio* Parametrization of Density Functional Dispersion Correction (DFT-D) for the 94 Elements H-Pu," The Journal of Chemical Physics 132 (2010): 154104, https://doi.org/10.1063/1.3382344.

(17) I. Soldo, P. Schweer, M. Quack, et al., "Ordered Chlorine Layer Formation from a Supersaturation of Chloroform," The Journal of Physical Chemistry C 128 (2024): 20053–20065, https://doi.org/10.1021/acs.jpcc.4c04678.
